# Transcriptome Analysis of Poplar during Leaf Spot Infection with *Sphaerulina* spp.

**DOI:** 10.1371/journal.pone.0138162

**Published:** 2015-09-17

**Authors:** Adam J. Foster, Gervais Pelletier, Philippe Tanguay, Armand Séguin

**Affiliations:** Canadian Forest Service, Natural Resources Canada, Laurentian Forestry Centre, Québec, Canada; McGill University, CANADA

## Abstract

Diseases of poplar caused by the native fungal pathogen *Sphaerulina musiva* and related species are of growing concern, particularly with the increasing interest in intensive poplar plantations to meet growing energy demands. *Sphaerulina musiva* is able to cause infection on leaves, resulting in defoliation and canker formation on stems. To gain a greater understanding of the different responses of poplar species to infection caused by the naturally co-evolved *Sphaerulina* species, RNA-seq was conducted on leaves of *Populus deltoides*, *P*. *balsamifera* and *P*. *tremuloides* infected with *S*. *musiva*, *S*. *populicola* and a new undescribed species, Ston1, respectively. The experiment was designed to contain the pathogen in a laboratory environment, while replicating disease development in commercial plantations. Following inoculation, trees were monitored for disease symptoms, pathogen growth and host responses. Genes involved in phenylpropanoid, terpenoid and flavonoid biosynthesis were generally upregulated in *P*. *balsamifera* and *P*. *tremuloides*, while cell wall modification appears to play an important role in the defense of *P*. *deltoides*. Poplar defensive genes were expressed early in *P*. *balsamifera* and *P*. *tremuloides*, but their expression was delayed in *P*. *deltoides*, which correlated with the rate of disease symptoms development. Also, severe infection in *P*. *balsamifera* led to leaf abscission. This data gives an insight into the large differences in timing and expression of genes between poplar species being attacked by their associated *Sphaerulina* pathogen.

## Introduction

There are numerous important diseases caused by members of the fungal family Mycosphaerellaceae that affect plants, and forest trees are no exception. Many *Populus* species and hybrids are susceptible to leaf and stem diseases caused by *Sphaerulina* species including leaf spot of balsam poplar (*P*. *balsamifera*), eastern cottonwood (*P*. *deltoides*) and black poplar (*P*. *nigra*) caused by *S*. *populicola*, *S*. *musiva* and *S*. *populi*, respectively, and stem cankers of hybrid poplars caused by *S*. *musiva*. Diseases caused by *S*. *musiva* were reported to reduce tree growth through premature defoliation during severe leaf spot infections [1–3]. Canker infections, caused by *S*. *musiva* are considered to be the most severe diseases in hybrid poplar plantations of North America [1–5]. Additionally, new and yet to be described species of Mycosphaerellaceae have been phylogenetically studied [6–8]. One of these non-described species, designated Ston1 was isolated from pycnidia emerging from leaf spots on *P*. *tremuloides* at Stoneham, Quebec, Canada, in 2003 ([6]; www.ncbi.nlm.nih.gov/biosample/SAMN02254960). Though not yet to be formally described, the genome of Ston1 was sequenced through the TAIGA (Tree Aggressors Identification using Genomic Approaches; www.taigaforesthealth.com) project and made publicly available (www.ncbi.nlm.nih.gov/genome/33152).

*Populus* spp. are an economically important group of tree species that are naturally distributed throughout the Northern Hemisphere. These trees have been traditional harvested for wood, pulp, paper and fuel. More recently, *Populus* spp. are being examined as a candidate source for new forms of bioenergy [9]. One species, *Populus trichocarpa*, serves as an important genetic model system for the study of trees [10] and was also the first tree species to have its genome sequenced [11].

The development of next-generation sequencing technologies such as Illumina, 454 Roche and Ion Proton provides excellent platforms for the study of gene expression through reverse-transcribed RNA sequencing (RNA-seq) [12]. RNA-seq has been used to study many biological systems in poplar, such as osmotic stress [13], wood development [14] and host-pathogen interactions. Detailed microarray and RNA-seq transcriptomic analysis have previously been reported on hybrid poplars infected with *Melampsora larici-populina* [15,16], *M*. *medusae* [15,17], and *Marssonina brunnea* [18] and in resistant and susceptible hybrids infected with *S*. *musiva* [19]. All of the reported transcriptomic studies focusing on host-pathogen interactions in poplar generated a wealth of information, including evidence on putative host defense genes and fungal virulence factors.

The study of the pathosystem between *Sphaerulina* and *Populus* could be established as a model system with many interesting and unique aspects. One such aspect is the study of molecular host-pathogen interactions during infection. To examine the basis of these interactions, we present a transcriptomic analysis of three *Populus* species during leaf infection by their co-evolved *Sphaerulina* pathogens. This approach differs from previous studies as it is not our goal to identify specific genes that allow a particular clone to be resistant to the pathogen, but instead to gain insight into the broad category of genes expressed in the different host species during infection. The specific interactions examined by RNA-seq are: *P*. *deltoides* infected with *S*. *musiva*, *P*. *balsamifera* infected with *S*. *populicola* and *P*. *tremuloides* infected Ston1.

## Material and Methods

### Plant and fungal material

Poplar stem sections were obtained on a public municipal park (Base de plein-air de Sainte-Foy, QC, Canada) with the permission of M. Jacques Grantham, director of the environment, Quebec City. These sampling activities did not involve endangered or protected species. Stem sections were taken from a single genotyped female tree of *P*. *deltoides* (BPSF– 073) and *P*. *balsamifera* (BPSF– 072) [20]. Clones were obtained by cutting stems into approximately 15-cm-long sections and placed in CleanStart Oasis Growing Medium No. 5015 (Oasis Grower Solutions, Kent, Ohio, USA). Cuttings were grown hydroponically with no nutrients or hormones added to the water in a greenhouse with light at 16 h day for 6 weeks. Rooted clones were then transferred to Pro-Mix BX (Premier Horticulture Inc., Rivière-du-Loup, Quebec, Canada) soil in 3 L pots and grown for a further 3 months before being transferred to growth chambers. Due to difficulty in obtaining a large number of evenly growing clones of *P*. *tremuloides* from root propagation, seeds from a single open pollinated female tree (#200010120) were obtained from the National Tree Seed Centre, Natural Resources Canada, Canadian Forest Service—Atlantic Forestry Centre, Fredericton, New Brunswick, Canada. Seeds were soaked in water for 24 h at 4°C prior to planting in high density trays filled with Pro-Mix BX. After 6 weeks, seedlings were transferred to 3 L pots and grown for a further 3 months in the greenhouse. The 5-month-old trees were transferred to Conviron growth chambers set at 22°C and 16 h light with a photosynthetic flux of 90–100 μmol m^-2^ s^-1^ and incubated for 4 weeks to reach a final age of 6 months.

One monosporal *Sphaerulina* isolate from each species was utilized. Isolates were prepared from spore suspensions stored in 25% glycerol at -80°C. All isolates were originally obtained from pycnidia emerging from leaf spots, but were collected at different locations and at different times: the *S*. *musiva* isolate (ND05.02) was originally obtained from a *P*. *deltoides* leaf in Notre-Dame du Nord, Quebec, Canada in 2002 [21]. *S*. *populicola* isolate 02.72A was isolated from *P*. *trichocarpa* in Marion County, Oregon, USA in 2002 [21] and the uncharacterized Ston1 isolate was obtained from leaf spots on *P*. *tremuloides* in Stoneham, Québec, Canada in 2003 [6]. All fungal isolates were confirmed to be members of their species by PCR-based detection [6,21]. To obtain conidia for foliar application, storage cultures were seeded to potato dextrose agar (PDA; Difco 213400) plates and grown at 22°C in the dark for 8 days. Conidia were harvested by flooding the plates (20 per isolate) with sterile distilled water, then filtered through 100-μl nylon mesh sterile Falcon® cell strainers (Fisher Scientific, Waltham, MA, USA), centrifuged at 4000 x *g* for 10 min, washed with 40 ml of sterile distilled water and resuspended in sterile distilled water to 1 x 10^5^ conidia L^-1^ prior to application.

### Experimental design and measurements

Eighteen clones for each tree species were prepared for experimentation. Trees from each species were separated into different growth chambers to prevent cross contamination by different *Sphaerulina* species. Using a Crown spray tool (North American Professional Products, Vaughan, Ontario, Canada), each tree was sprayed with 10 mL of atomized spore suspension (1 x 10^5^ conidia mL^-1^). Three trees from each species were sprayed with water to serve as controls. To further prevent spore cross contamination, inoculation of each species was done on separate days. The leaves from each tree were harvested following a timeline such that trees were only sampled once. The top three fully expanded leaves were excised, pooled and flash frozen in liquid nitrogen for storage prior to DNA and RNA extraction. Samples were taken 0, 1, 4, 8, 15 and 30 days after inoculation (DAI). All leaves on trees were monitored daily for signs of disease such as the occurrence of leaf spots, leaf abscission and plant height. Disease was scored based on a four-point scoring system for leaf spots on each tree prior to harvest, where 1 represents 1–5% of leaf area, 2 represents 6–30% of leaf area and 3 represents over 30% of leaf area. A score of 0 was assigned when no leaf disease symptoms were observed. Differences in plant height and in the number of leaves were statistically analyzed at 35 DAI by analysis of variance (ANOVA) and Tukey’s pair-wise HSD test with JMP 8.0 (SAS Institute Inc., Cary, North Carolina, USA).

### DNA and RNA extraction

Both DNA and RNA were extracted from the leaves following inoculation. DNA was used to track the growth of the fungi through quantitative PCR (qPCR), while RNA was used for transcriptome analysis and reverse transcript-qPCR (RT-qPCR). Leaves were homogenized in a liquid nitrogen chilled mortar and pestle, and were then stored at -80°C to be used for both RNA and DNA extraction. DNA was isolated from 100 mg of tissue for each sample using the DNeasy Plant Mini Kit (Qiagen, Toronto, Ontario, Canada). RNA was also isolated from 100 mg of tissue using the RNeasy Plant Mini Kit (Qiagen) with in-column RNase-Free DNase Set (Qiagen) treatment.

### Transcriptome sequencing

RNA was extracted from each tree sampled and assayed for quality with a 2100 Bioanalyzer (Agilent, Santa Clara, California, USA). All RNA samples had a ratio of absorbance at 260 and 280nm (A260/280) above 2.0 and no peaks indicating the presence of genomic DNA were detected. When further analyzed using a 2100 Bioanalyzer, all samples had a RNA integrity number (RIN) greater than 7. Libraries for sequencing were prepared by Brian Boyle at the Institut de biologie intégrative et des systèmes (IBIS) of Université de Laval, Quebec, Quebec, Canada. Each cDNA library was barcoded and six samples were pooled together. In total, six lanes of pooled samples were single-end sequenced using an Illumina HiSeq 2500 platform at the McGill University and Génome Québec Innovation Centre (Montreal, Quebec, Canada).

### RNA-seq pipeline

The sequencing libraries obtained from Génome Québec were analyzed using RobiNA software version 1.2.4 (build656) pipeline [22]. First, the data was quality checked for base call quality, homopolymers, kmer frequency, base call frequency and overrepresented sequences. Data was then trimmed by removing the adapter sequences, low quality reads as well as reads shorter than 20 base pairs. The data were then aligned separately to the *P*. *trichocarpa* V3.0 210 genome build using BOWTIE [23] set to a seed length of 28 base pairs allowing up to two mismatches. Unaligned reads were saved for further analysis. The mapped libraries were then analyzed for digital gene expression (DGE) using edgeR [24] with a Benjamini—Hochberg correction and false discovery rate (FDR) threshold of 0.05.

Gene annotations for significantly expressed poplar genes with a log2 fold change > 1/-1 were obtained from the *P*. *trichocarpa* V3.0 210 peptide data set. Significantly expressed poplar genes were functionally annotated using BLAST2GO version 2.8 [25]. Fisher’s exact test was performed on a list of significantly differentially expressed genes at each time point with a log2 fold changes > 2.0. GO terms were screened to generate the most specific terms with a FDR < 0.05. Benjamini-Hochberg multiple testing correction of FDR was also performed. MapMan version 3.5.1 was used to display differentially expressed poplar genes (log2 fold change > 1/-1) according to their metabolic pathways. [26]. The Wilcoxon rank sum test was used to identify groups of genes in specific functional bins whose gene expression had greater variation then the entire dataset [26].

Raw RNA-seq files as well as read count and digital gene expression analysis matrixes for *P*. *trichocarpa* primary genes are publicly available on NCBI Gene Expression Omnibus (GEO) (accession number: GSE67697).

### Quantitative PCR

To determine the relative abundance of fungal DNA within the leaf samples, DNA was extracted from the leaves of inoculated plants and qPCR was used to amplify and determine the abundance of the actin-1 fungal gene (*S*. *musiva* transcript 147624, *S*. *populicola* transcript 133327 and Ston1 NCBI genome: 33152, Contig00301:46340–46570) relative to poplar α-tubulin-1 gene (Potri.002G111900.1). The Ston1 actin-1 gene was identified as the top BLASTN match to *S*. *musiva* transcript 147624. Differences in relative fungal abundance between time points was statistically analyzed by ANOVA and Tukey’s pair-wise HSD test.

RT-qPCR was used to validate the expression profile of 13 poplar genes identified by the RNA-seq analyses. Ten genes with induced expression in one or more of the host-pathogen systems following inoculation were chosen, while three genes with stable expression were chosen to represent non-induced reference genes. Prior to RT-qPCR, cDNA libraries were generated from each RNA sample using the QuantiTect Reverse Transcription Kit (Qiagen).

Primers were designed using the Oligo Analyzer/Oligo Explorer program (http://www.genelink.com/tools/gl-oe.asp). Annealing temperature (Tm) values were set at 65°C using the following parameters: 50 mM NaCl concentration and 250 pM DNA. No template control reactions were run on the primer pairs to detect dimer formation. A list of primers used in this study is shown in S1 Table.

Gene expression was analyzed for each of the sample using the Light Cycler 480 (Roche). All reactions were performed in a final volume of 10 μl and contained 1X QuantiTect SYBR® Green PCR Master Mix (Qiagen), 0.5 μM of each primer, and 1 μl of template DNA, which is equivalent to 10 ng of total RNA. PCR thermocycling conditions were set at 95°C for 15 min, 50 cycles at 95°C for 15 sec, 58°C for 30 sec, and 65°C for 90 sec. Fluorescent readings were taken at the end of each cycle, and the specificity of amplification as well as the absence of primer dimers were confirmed with a melting curve analysis at the end of each reaction. To correct for technical variation in RNA extraction, total RNA quantification, reverse transcription, and RT-qPCR reactions as well as biological variation expression data were normalized against the geometric mean of three reference genes, UBi 10 (Potri.014G115100.1), Eif 4 (Potri.006G225700.1), and MKK 2 (Potri.018G050800.1), by geNORM VBA applet for Microsoft Excel (http://medgen.ugent.be/jvdesomp/genorm) [27]. For all experiments, fold change is expressed as treatment relative to the control calculated using the 2^-ΔΔCt^ method [28]. Standard deviation related to the biological variation within one line was calculated in accordance with error propagation rules. Statistical analysis of gene expression between 1 and 15 DAI was conducted using Student’s paired t-test at p < 0.05.

## Results

RNA-seq was successfully used to associate disease symptoms with molecular plant responses. Large differences were observed between the different plant pathogen interactions regarding the rate of disease development, observed phenotypes during infection and molecular pathways significantly regulated during infection.

### Disease development

**Fig 1:**
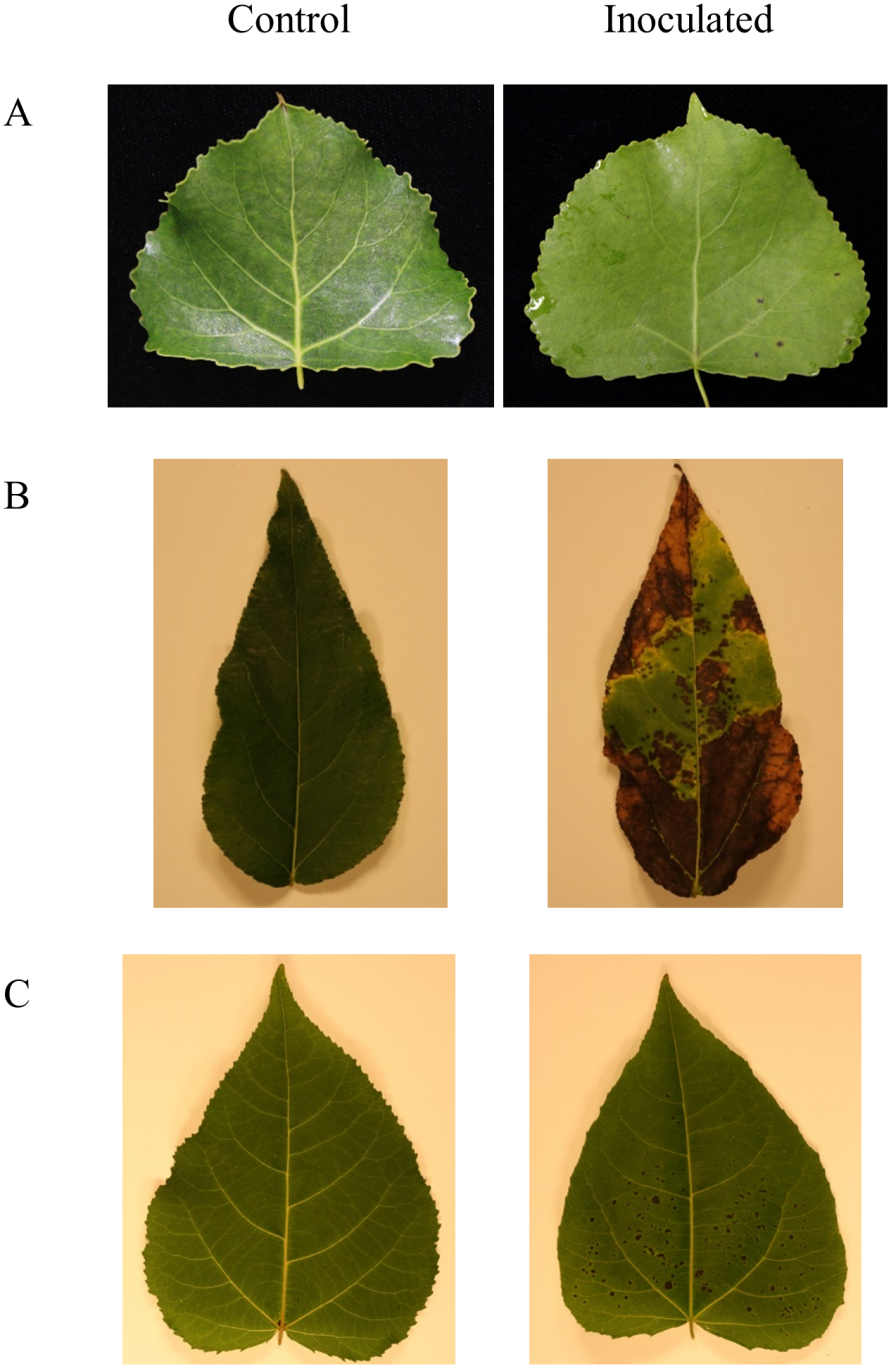
Control and inoculated leaves at 30 DAI. Pictures show the adaxial surface of a fourth fully expanded leaf at time of inoculation. (A) *P*. *deltoides* inoculated with *S*. *musiva*. (B) *P*. *balsamifera* inoculated with *S*. *populicola*. (C) *P*. *tremuloides* inoculated with Ston1.

**Fig 2:**
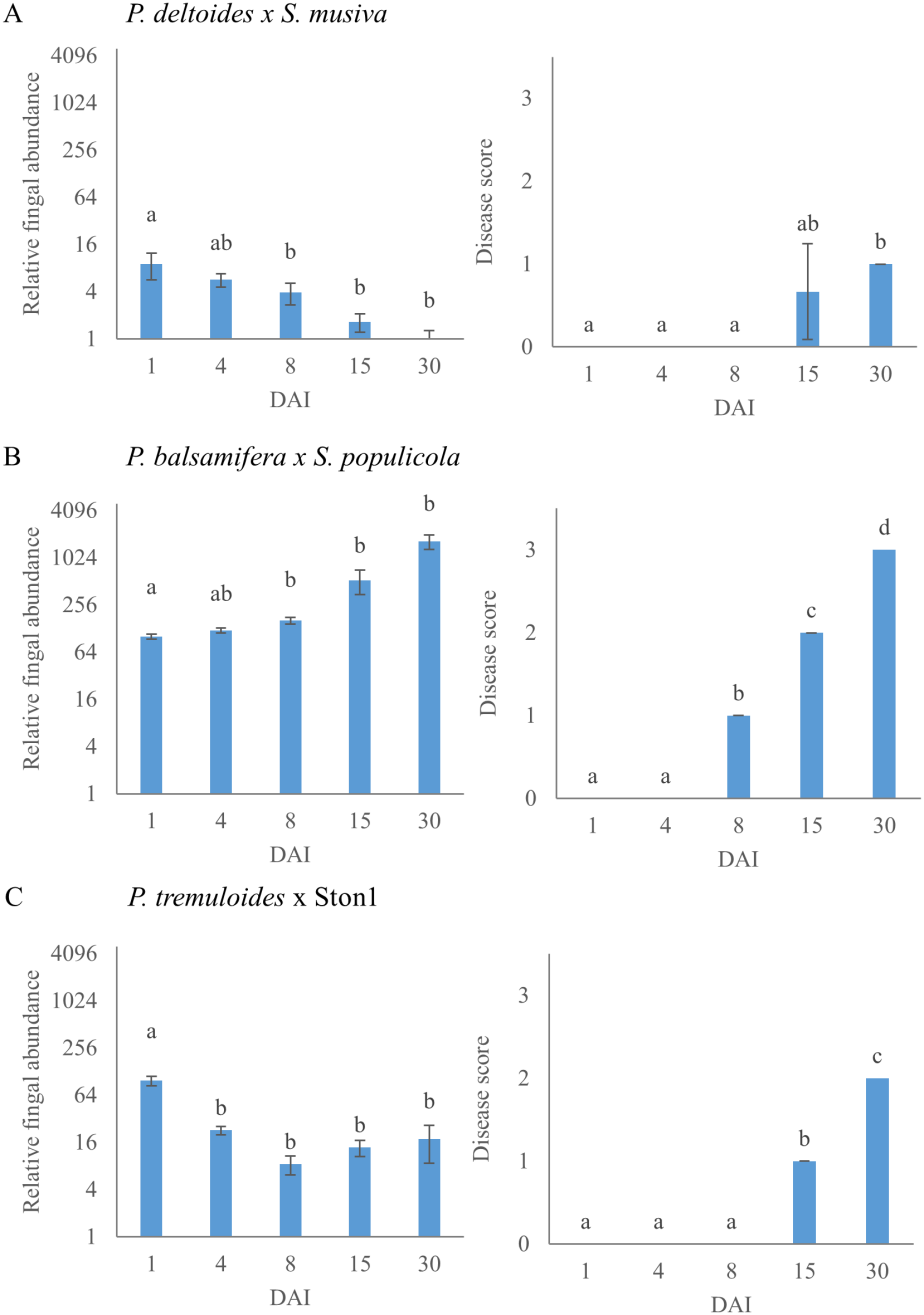
Pathogen relative fungal abundance and disease severity scores in each pathosystem. (A) *P*. *deltoides* inoculated with *S*. *musiva*. (B) *P*. *balsamifera* inoculated with *S*. *populicola*. (C) *P*. *tremuloides* inoculated with Ston1. Fungal DNA abundance relative to plant DNA measured by qPCR shown in log scale. Each data point represents the mean value of 3 samples. Bars with the same letter are not significant at p < 0.05 using ANOVA and Tukey’s HSD test.

Leaf spots developed in all poplar-*Sphaerulina* pathosystems, but with distinct temporal and spatial patterns within individual trees and between tree host species. Leaf spots occurred earlier and more severely on lower, more mature leaves after inoculation. During inoculation, stems were also coated in atomized conidia; however, no stem disease was observed in any pathosystem. The level of disease severity between the pathosystems was different. Fig 1 shows both healthy non-inoculated leaf and an infected leaf 30 DAI. The occurence of first disease symptoms varied between pathosystems. In *P*. *deltoides*, leaf spots were first observed 14 DAI as minute localized discolouration of the epidermis on the adaxial leaf surface. In *P*. *balsamifera* and *P*. *tremuloides*, the visual symptoms appeared earlier, at 8 and 9 DAI, respectively. The disease severity scores were relatively low for *P*. *deltoides* with 1 and *P*. *tremuloides* with 2. Leaf spot symptoms were much greater in *P*. *balsamifera* and disease severity scores reached the maximum on the four-point scale (Fig 2). Pathogen growth was quantified by qPCR using genomic DNA extracted from infected leaves (Fig 2). In *P*. *deltoides* and *P*. *tremuloides*, the amount of fungal DNA significantly decreased from the initial spore application at later time points (Fig 2). In *P*. *balsamifera*, pathogen growth did not significantly increase from 1 to 15 DAI, but significantly increased by 30 DAI (Fig 2).

**Fig 3:**
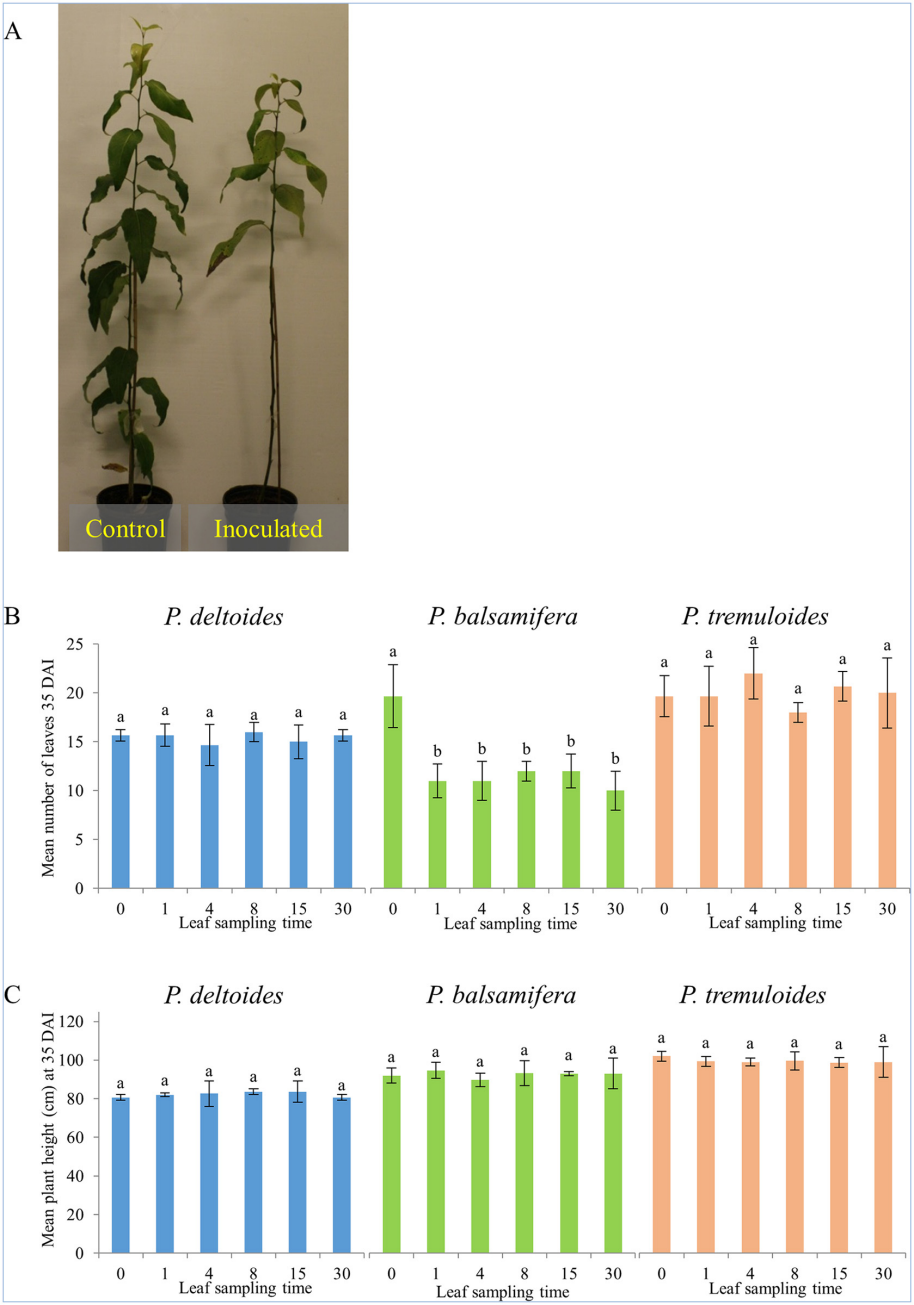
Leaf abscission in *P*. *balsamifera* in response to inoculation with *S*. *populicola* at 35 DAI. (A) Control and inoculated trees at 35 DAI. (B) Number of leaves of control and inoculated trees at 35 DAI grouped by leaf harvest time point. (C) Plant height of control and inoculated trees at 35 DAI grouped by leaf harvest time point. Mock indicates non-inoculated control trees at 0 DAI. Error bars show standard deviation. Bars with the same letter are not significant at p < 0.05 using ANOVA and Tukey’s HSD test.

The lowest leaves on all inoculated *P*. *balsamifera* clones began to abscise at 20 DAI, which was 12 days after appearance of the first leaf spots. By 35 DAI, the majority of leaves on the inoculated plants had abscised (Fig 3A). At 35 DAI, there was a significant difference in the number of leaves in the control vs inoculated plants (Fig 3B). No significant leaf abscission was observed in the other two pathosystems. No statistical differences in plant height at 35 DAI were detected between inoculated and controls plants in all tree species (Fig 3C). Leaf abscission appears to have had no effect on plant growth, but this may be due to the short nature of this 35 day study. The timing of leaf harvest had no significant effect on leaf number or plant height at 35 DAI.

### Mapping statistics of transcriptome analysis

In total, over 100 billion base pair (bp) of sequenced information in 1.06 billion 100 bp reads was obtained from all combined libraries (Table 1). Each individual library ranged from 9.5–46 million reads. These reads were mapped to the *P*. *trichocarpa* genome version 3. Summary mapping statistics for each time point in each pathosystem are summarized in Table 1, and each individual library is shown in S2 Table. A significant difference in read mapping was observed between pairwise analyses of read mapping from each poplar species. Reads generated from *P*. *balsamifera* had the highest percentage of mapping to the poplar genome at 55.5%, followed by 53.5% for *P*. *deltoides* and 42.6% for *P*. *tremuloides*. As expected, read mapping percentage was highest with the closest related poplar species, *P*. *balsamifera* and lowest with the more distantly related species to *P*. *trichocarpa* [29]. Mapping variation between biological replicates in each pathosystem is shown in S1 Fig, where the lowest variation between biological replicates at each time point was observed in *P*. *balsamifera*. An average of 24947 ± 720 transcripts were mapped with a minimum sum of 10 reads from replicates for all species and time points with no significant variation in the number of genes. The poplar genome is known to be highly heterozygous [30], and these variations could also have an effect on read mapping.

**Table 1:** Statistical summary of read mapping to the *P*. *trichocarpa* genome.

Plant	Fungus	DAI	Total reads	Mapped	% Mapped	# genes^a^
*P*. *deltoides*	*S*. *musiva*	0	114054743	61834026	54.20%	25226
*P*. *deltoides*	*S*. *musiva*	1	92102107	49356037	53.60%	24274
*P*. *deltoides*	*S*. *musiva*	4	113588213	61791380	54.40%	24815
*P*. *deltoides*	*S*. *musiva*	15	87570660	45512697	52.00%	24119
*P*. *balsamifera*	*S*. *populicola*	0	82163878	46666817	56.80%	25228
*P*. *balsamifera*	*S*. *populicola*	1	78255302	43586856	55.70%	24772
*P*. *balsamifera*	*S*. *populicola*	4	87779536	48103625	54.80%	25685
*P*. *balsamifera*	*S*. *populicola*	15	100033038	54822460	54.80%	25694
*P*. *tremuloides*	Ston1	0	90444097	45537234	50.30%	25573
*P*. *tremuloides*	Ston1	1	81965142	39237333	47.90%	25543
*P*. *tremuloides*	Ston1	4	57930670	22544200	38.90%	23366
*P*. *tremuloides*	Ston1	15	73764072	24634259	33.40%	25072

^a^Total number of genes identified with a minimum sum of 10 reads between biological replicates.

Two individual libraries from *P*. *tremuloides* had poor read mapping statistics, with only 33.1% and 7.5% of reads successfully mapping at 4 DAI and 15 DAI, respectively. These two libraries both had consistent base quality scores above 30 for the majority of reads; however, a kmer enrichment test revealed different conserved 5 bp sequences present at multiple locations reaching an enrichment factor up to 10%. Due to this, potential library construction or sequencing error resulted in 40% and 80% of the reads in one sample at 4 DAI and 15 DAI to be excluded from mapping. Despite the reduction of raw reads, 12 million and 1.8 million reads were still successfully and accurately mapped. These datasets were included in the downstream statistical analysis, though this resulted in a decreased confidence in gene detection due to the substantially variation in read depth (S2 Table).

### Differential gene expression analysis

Species and time specific sets of genes are differentially expressed in each pathosystem. In total, over 5000 poplar genes were identified with differential expression between inoculated and healthy tissues. Contrasts for poplar gene mapping were conducted between non-inoculated healthy plants and infected plants at 1, 4 and 15 DAI. The Venn diagrams shown in S2 and S3 Figs demonstrate the unique and shared differentially expressed genes at each time point and between each pathosystem. In all poplar species, the number of observed differentially expressed genes was higher later during infection (S3 Fig). The number of differentially expressed genes identified was higher at 4 DAI than at 1 DAI in *P*. *tremuloides*; however, in the other two plant species, there were fewer differentially expressed genes observed at this midpoint. At 15 DAI, over 1000 upregulated and downregulated genes were identified at each time point and significantly regulated genes were shared between different time points. A number of genes were commonly differentially expressed between host species at various time points (S3 Fig).

### Functional annotation of differentially expressed genes

**Fig 4:**
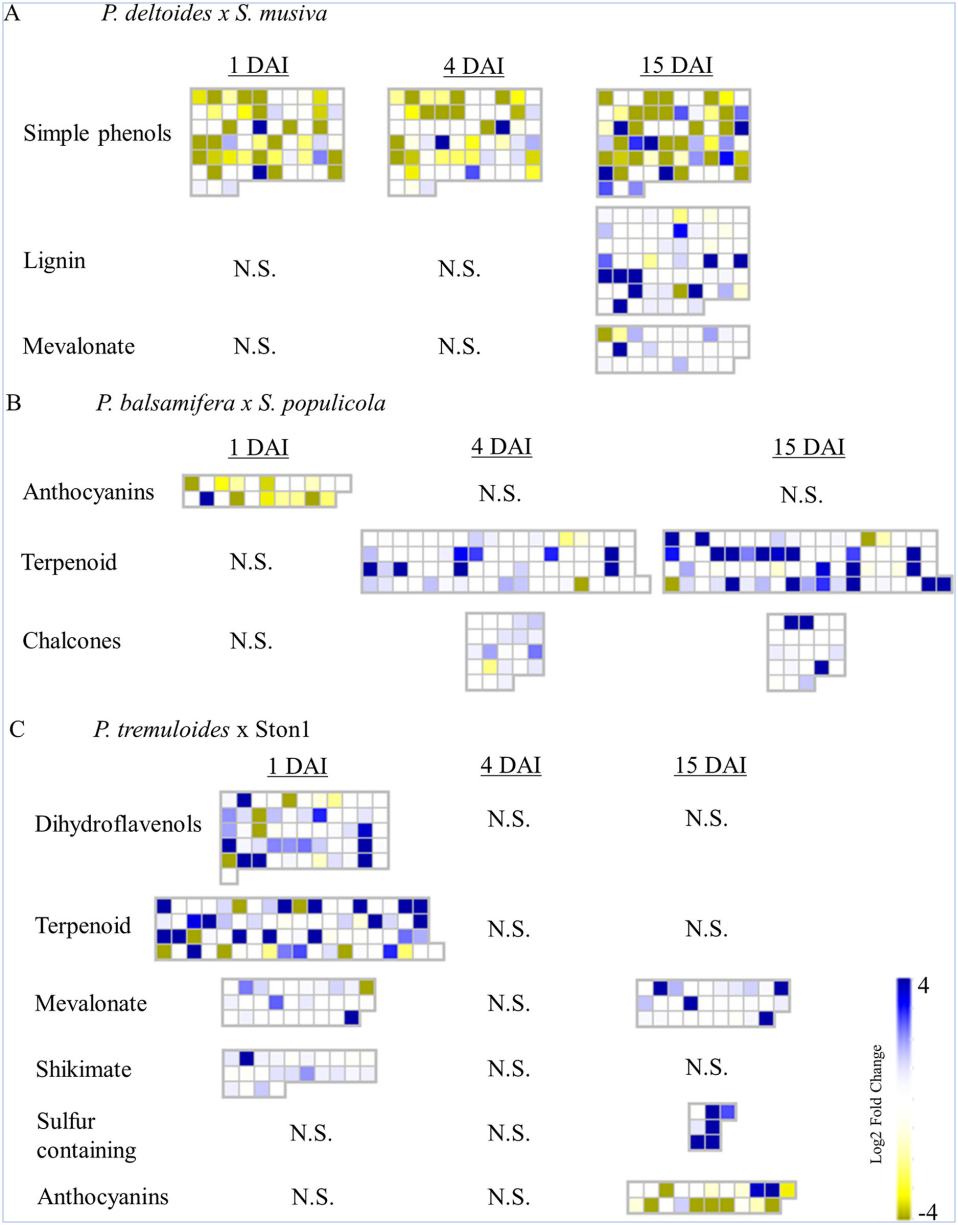
Differentially regulated secondary metabolite pathways expressed at different time points. (A) *P*. *deltoides* infected with *S*. *musiva*, (B) *P*. *balsamifera* infected with *S*. *populicola* and (C) *P*. *tremuloides* infected with Ston1. Log2 fold ratios are represented by a colour gradient, with blue being upregulated and yellow being downregulated. The pathways shown have a p < 0.05 after Benjamini-Hochberg correction. N.S. are not significantly regulated. Partial heat map shown for lignin biosynthesis pathway.

Different functionally annotated genes and gene families were expressed at different time points between the three pathosystems. Pathway analysis focused on the examination of secondary metabolic pathways to assess the major host defensive responses. MapMan graphical secondary metabolic pathway was used to assess the plant response to fungal inoculation (Table 2). Wilcoxon rank sum test was used to identify significantly regulated pathways at each time point. Gene expression data for each significantly regulated pathway are shown in the heat maps of Fig 4 for each species, where each box represents a gene annotating to the specific bin ID to the given pathway. Only genes that annotated to MapMan secondary metabolite bins are shown and only a representative fraction of lignin biosynthesis genes are shown. Overall, genes involved with anthocyanin and simple phenol biosynthesis are generally downregulated, while genes of the terpenoid, lignin or flavonoid biosynthesis are generally upregulated. In *P*. *deltoides*, which showed the lowest level of leaf spot infection, the significant regulation in simple phenol biosynthesis pathways was the only observed change occurring before leaf spot emergence. After leaf spot formation at 15 DAI, genes involved in lignin biosynthesis were observed (Table 2).

**Table 2:** Differentially expressed secondary metabolism pathways between control and inoculated poplar trees during infection with *Sphaerulina* spp.

		*P*. *tremuloides* x Ston1			*P*. *balsamifera* x *S*. *populicola*			*P*. *deltoides* x *S*. *musiva*		
Secondary Metabolism Pathway	MapMan bin	1 DAI	4 DAI	15 DAI	1 DAI	4 DAI	15 DAI	1 DAI	4 DAI	15 DAI
Shikimate pathway	13.1.6.1	**0.10**	0.20	0.35	0.25	0.73	0.25	0.70	0.23	0.95
Mevalonate pathway	16.1.2	**0.09**	0.66	**0.08**	0.25	0.21	0.13	0.11	0.25	**0.09**
Terpenoid biosynthesis	16.1.5	**0.02**	0.72	0.88	0.15	**0.08**	**2.5E-03**	0.64	0.71	0.64
Simple phenols	16.10	0.31	0.11	0.82	0.15	0.27	0.11	**9.9E-04**	**0.01**	**0.08**
Phenylpropanoids	16.2	0.16	0.93	0.92	**0.05**	0.80	0.58	1.00	0.63	0.58
Lignin biosynthesis	16.2.1	0.49	0.72	0.66	0.23	0.93	0.95	0.75	0.76	**0.01**
Sulfur-compound biosynthesis	16.5.99	0.46	0.92	**0.03**	0.88	0.88	0.91	0.83	0.57	0.86
Flavonoid anthocyanins	16.8.1	0.36	0.71	**0.07**	**0.01**	0.34	**0.06**	0.67	0.44	0.58
Flavonoids chalcones	16.8.2	0.13	0.29	0.97	0.99	**0.09**	0.25	0.74	0.69	0.67
Flavonoids dihydroflavonols	16.8.3	**0.01**	0.61	0.89	0.65	0.56	0.74	0.83	0.96	0.74

Analysis performed using Wilcoxon rank sum test with Benjamini-Hochberg correction at p < 0.1. DAI = days after inoculation; P-values in bold are significant at p ≤ 0.1

In the more moderately infected *P*. *tremuloides* trees, terpenoid biosynthesis genes, including those in the mevalonate pathway that produces the isoprenoid precursors needed for terpenoid biosynthesis, were upregulated at 1 DAI (Table 2). However, these pathways were no longer observed to be differentially regulated midway through infection at 4 DAI, when fungal biomass was decreasing. After leaf spot formation, the mevalonate pathway was again activated along with the biosynthesis of sulfur containing secondary metabolites, while genes involved in anthocyanin biosynthesis were generally downregulated.

Terpenoid biosynthesis gene upregulation was also observed in the *P*. *balsamifera* clones, but with a different timing of expression than in *P*. *tremuloides*. The terpenoid pathway was differentially regulated at 4 DAI until leaf spot formation, but was not observed early during infection when only the general downregulation of anthocyanin biosynthesis was observed (Table 2). Also, flavonoid chalcone biosynthesis was significantly regulated both before and after leaf spot formation in correlation with terpenoid biosynthesis. *P*. *balsamifera* clones were highly susceptible to leaf spot despite the expression of these genes; therefore, the differential expression of genes involved in terpenoid and anthocyanin biosynthesis may indicate an insufficient defense response to *S*. *populicola*.

Fisher’s exact test was used successfully to identify specific GO terms at different time points during leaf spot infection. Similar results were observed between MapMan and BLAST2GO analysis. No specific GO terms were overrepresented in *P*. *deltoides* at 1 DAI or 4 DAI. At 15 DAI, a number of GO terms involved in active defense responses to fungal pathogens were detected (S4 Fig). Significantly overrepresented GO terms were identified at 1 DAI and 15 DAI in *P*. *balsamifera* (S5 Fig). In *P*. *tremuloides*, overrepresented GO terms were identified for all time points examined (S6 Fig). At 15 DAI, a large number of overrepresented GO terms were identified in all three poplar species (S4–S6 Figs). The respective pathogen appeared to be detected soon after inoculation in *P*. *tremuloides* and *P*. *balsamifera*, as a number of GO terms associated with defense responses to fungi, innate immune responses, defense involving deposition of callose, oxidation-reduction processes and transcription factors are overrepresented at 1 DAI. Few or no GO terms were observed to be overrepresented at 4 DAI in all pathosystems. However, at 15 DAI, all poplar species were expressing a number of fungal defensive response reactions such as PR4-like endochitinase, lignin biosynthesis protein, and CC-NBS-LRR and TMV resistance protein encoding genes.

GO terms associated with hormone signaling were also overrepresented in all species at 15 DAI. The ethylene-mediated signaling pathway was overrepresented in *P*. *deltoides* (S4 Fig), salicylic acid and jasmonic acid signaling pathways were overrepresented in *P*. *balsamifera* (S5 Fig), while jasmonic acid, salicylic acid and abscisic acid signaling pathways were overrepresented in *P*. *tremuloides* (S6 Fig).

### Gene expression related to visible phenotypes and defense

**Fig 5:**
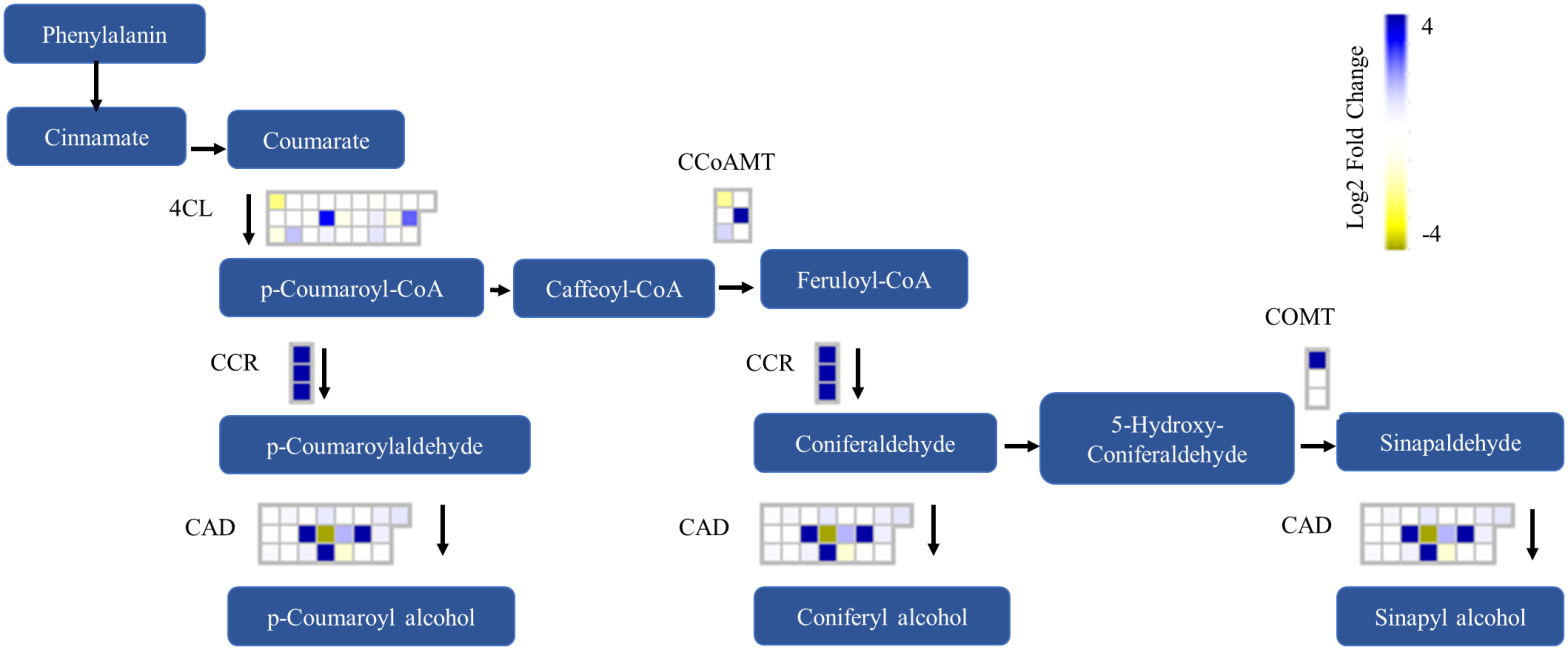
Lignin biosynthesis pathway with heat maps showing *P*. *deltoides* gene expression in response to *S*. *musiva* at 15 DAI. Log2 fold ratios are represented by a colour gradient, with blue being upregulated and yellow being downregulated. Graphic regenerated from MapMan 3.5.1R2 lignin synthesis.png mapped to *P*. *trichocarpa* v3.0_210_peptide.m02. 4CL, 4 coumaroyl-CoA ligase; CCoAMT, caffeoyl-CoA O-methyltransferase; CCR, cinnamoyl-CoA reductase; COMT, caffeate O-methyltransferase; CAD, cinnamoyl alcohol dehydrogenase.

Two major phenotypes were observed in poplar following infection. A more precise examination of gene expression reveals that multiple lignin biosynthesis genes of *P*. *deltoides* are significantly upregulated after leaf spot formation (Fig 5). Among these genes were a single caffeoyl-CoA 3-O-methyltransferase (CCoAMT) that was more than 6 log2-fold upregulated, three cinnamoyl CoA reductases (CCR) were more than 4 log2-fold upregulated and three cinnamyl-alcohol dehydrogenases (CAD) that were more than 4 log2-fold upregulated. The highest overexpressed CAD gene, potri.009g062800.1, shares close similarity with the defense-related *A*. *thaliana* ELI3-2 gene [31].

Another observable phenotype was the senescence and abscission of leaves in *P*. *balsamifera* following infection with *S*. *populicola* (Fig 3). To determine an underlying molecular cause for this phenotype, gene expression data was screened for the presence of annotations associated with leaf abscission and senescence. A small subset of genes putatively involved in leaf senescence were overexpressed in *P*. *balsamifera* at 4 DAI (Table 3) prior to visible disease symptoms. By 15 DAI, two additional senescence-related genes were significantly overexpressed: Potri.001G112600 and Potri.010G166200. These were significantly overexpressed in all three species at 15 DAI compared to the control. In this study, only leaf tissue was examined and petioles were excluded; thus, abscission zone tissue-specific genes were not screened.

**Table 3:** Expression of genes putatively involved in leaf senescence and abscission.

	*P*. *deltoides*			*P*. *balsamifera*			*P*. *tremuloides*			
Gene ID	1 DAI	4 DAI	15 DAI	1 DAI	4 Dai	15 DAI	1 DAI	4 DAI	15 DAI	Description
Potri.011G040500	-0.34	-0.34	2.04	5.61	**8.54**	**7.74**	1.82	1.22	0.98	d6-type cyclin
Potri.005G098200	0.00	0.00	**8.69**	4.57	**3.59**	**5.68**	1.63	1.42	3.10	nac domain protein
Potri.005G251300	0.19	-0.13	0.87	3.93	**3.45**	**3.81**	0.45	0.69	0.88	btb poz domain protein
Potri.010G166200	2.14	2.99	**6.07**	4.13	**3.11**	**3.71**	1.43	0.33	**2.31**	nac domain protein
Potri.015G020000	-0.68	3.47	0.73	0.04	1.46	**3.19**	-0.42	0.33	**2.40**	nac domain ipr003441
Potri.012G091400	-0.47	-7.73	0.40	1.93	**3.29**	**3.12**	0.35	0.59	0.57	btb poz and taz domain protein
Potri.001G148700	-0.25	0.09	0.55	1.97	**1.97**	**2.56**	-0.35	-0.06	0.98	senescence-associated family protein
Potri.001G112600	1.92	1.02	**1.99**	0.44	0.26	**1.74**	-0.08	0.11	**2.01**	senescence-inducible stay-green protein

Genes shown have a minimum of 2log fold change between 1 DAI and 0 DAI. Numbers in bold show statistical significance at FDR < 0.05. Only genes with senescence or abscission GO terms that were significantly overexpressed at 15 DAI in *P*. *balsamifera* are shown.

Upregulation of genes with annotation relating to plant defense were detected at 1 DAI. Among these genes upregulated were chitinases, β-glucosidases and NBS-LRR resistance proteins (Table 4). Chitinase activity was the most common defensive function observed at 1 DAI, representing 8 of 37 shared upregulated defensive genes, though no individual chitinase genes were commonly regulated between pathosystems. Two β-glucosidases were commonly upregulated at 1 DAI between *P*. *deltoides* and *P*. *balsamifera*. After 1 DAI, those genes were no longer significantly upregulated in *P*. *deltoides*, but were upregulated in *P*. *balsamifera* throughout the entire time course. In *P*. *tremuloides*, one β-glucosidase (Potri.004G110200) was significantly upregulated at 4 DAI, and it remained upregulated at 15 DAI.

**Table 4:** Significantly expressed genes at 1 DAI that have GO annotations to defense.

Gene ID	*P*. *deltoides*	*P*. *balsamifera*	*P*. *tremuloides*	Description
Potri.019G010800	**2.1**	**4.0**	0.0	β-glucosidase
Potri.004G110200	**2.3**	**2.9**	0.0	linamarase family protein
Potri.006G188300	**2.7**	-1.3	-0.4	class v chitinase
Potri.001G104500	-0.1	**2.1**	-0.4	gamma-glutamylcysteine synthetase
Potri.001G107600	0.7	**2.3**	-0.4	osmotin precursor
Potri.002G047500	-0.4	**2.6**	0.0	calcium-transporting atpase
Potri.003G069300	-0.1	**2.7**	0.1	glycolate oxidase
Potri.003G071400	0.3	**2.5**	0.4	zinc finger
Potri.004G050000	0.3	**3.7**	0.9	mlo-like protein
Potri.006G127200	0.1	**2.2**	-1.1	rna-binding protein
Potri.006G251700	0.0	**2.8**	-0.8	overexpressor of cationic peroxidase
Potri.008G213100	1.5	**2.5**	-0.3	Bet v I family pathogenesis-related protein
Potri.009G108200	0.8	**2.5**	-1.3	maternal effect embryo arrest 14 protein
Potri.011G021100	0.3	**2.1**	0.0	probable tyrosine-protein phosphatase
Potri.011G105400	0.0	**3.0**	-0.1	probable salt tolerance-like protein
Potri.013G098900	1.3	**3.2**	0.7	leucine-rich repeat family protein
Potri.013G125000	0.5	**2.7**	1.7	class iv chitinase
Potri.017G079500	2.2	**3.6**	2.4	probable wrky transcription factor 72-like
Potri.019G093700	0.6	**3.3**	**5.2**	endochitinase pr4-like
Potri.001G014200	0.0	0.3	**3.3**	transposon en spm sub-class
Potri.001G425300	0.6	-0.3	**3.1**	cc-nbs-lrr resistance protein
Potri.001G427700	0.1	-0.4	**2.1**	disease resistance gene nbs-lrr family protein
Potri.004G182100	4.6	0.0	**7.2**	class i chitinase
Potri.004G196100	-0.2	-1.8	**3.1**	lrr and nb-arc domains-containing disease resistance
Potri.006G094400	0.3	0.0	**3.1**	fructose-bisphosphatase
Potri.009G141800	0.8	1.5	**6.8**	acidic four domain chitinase
Potri.009G142000	4.6	4.6	**5.8**	acidic four domain chitinase
Potri.009G142100	1.5	-4.4	**6.1**	acidic four domain chitinase
Potri.009G142300	0.0	-0.1	**4.1**	acidic four domain chitinase
Potri.010G151200	0.0	0.0	**5.9**	pop3 peptide
Potri.011G012900	0.0	-0.4	**2.7**	tir-nbs type disease resistance protein
Potri.013G041900	-0.9	0.3	**3.2**	wound-induced protein win2
Potri.013G133100	0.9	0.8	**5.7**	lrr receptor-like protein kinase
Potri.014G046800	0.0	0.0	**7.6**	ap2 erf domain-containing transcription factor
Potri.015G126100	0.1	-1.7	**8.1**	calmodulin-binding protein
Potri.019G052000	0.0	-2.5	**2.5**	tir-nbs disease resistance-like protein
Potri.T014400	-0.8	0.2	**4.2**	bed finger-nbs-lrr resistance protein

Numbers in bold show statistical significance at FDR < 0.05.

The expression of a group of plant proteases belonging to the Kunitz-type were significantly regulated in all pathosystems (Table 5). In *P*. *deltoides*, one upregulated and two downregulated Kunitz trypsin inhibitors were detected at 15 DAI. In *P*. *balsamifera*, three Kunitz trypsin inhibitors were observed upregulated early during infection, but none were upregulated at 4 DAI. At 15 DAI, 15 genes belonging to this protease family were overexpressed. In *P*. *tremuloides*, four or more Kunitz trypsin inhibitors were upregulated at all of the sampled time points.

**Table 5:** Selection of poplar Kunitz-type trypsin inhibitor and WRKY family genes expressed in each poplar species following inoculation with *Sphaerulina* spp.

		*P*. *deltoides*			*P*. *balsamifera*			*P*. *tremuloides*		
Gene ID	Gene Family	1 DAI	4 DAI	15 DAI	1 DAI	4 DAI	15 DAI	1 DAI	4 Dai	15 DAI
Potri.001G309900	KTI	0.41	1.46	1.39	0.36	1.43	**2.54**	0.17	1.42	**7.48**
Potri.003G097900	KTI	-3.59	-1.88	**-8.22**	0	0	0	0.39	0.87	**-5.03**
Potri.004G000400	KTI	0.12	1.27	**2.77**	-0.53	0.58	**2.07**	-1.52	**-10.33**	**4.34**
Potri.004G067800	KTI	1.77	1.21	1.96	-1.2	-0.42	1.67	2.22	**2.27**	**5.84**
Potri.004G067900	KTI	0	0	4.52	**-2.01**	**-2.94**	**-3.16**	0.83	-0.04	**-3.5**
Potri.007G111500	KTI	-0.62	-2.28	**-3.75**	0.66	2.22	**3.11**	-1.54	**-9.4**	-1.27
Potri.007G111600	KTI	-0.68	0.09	0.92	0.91	1.77	**2.95**	0.19	-2.06	-1.68
Potri.007G111700	KTI	0.33	0.26	0.63	-0.43	1.01	**3.21**	7.03	0	0
Potri.010G007900	KTI	0	0	0	6.6	0	4.24	**4.73**	1.02	1.43
Potri.019G011000	KTI	1.34	1.05	1.21	0	4.45	6.2	-0.79	**4.04**	-5.55
Potri.019G088200	KTI	-6.56	-6.56	-2.01	**8.06**	0	0	**3.62**	-8.38	**-8.38**
Potri.019G124400	KTI	0.87	0.01	1.48	0.78	-1.7	**-1.99**	**6.84**	**4.2**	**2.96**
Potri.019G124600	KTI	1.97	0.4	1.68	0.56	-0.32	-1.02	**5.29**	**2.65**	1.01
Potri.019G124700	KTI	-4.28	0.04	-4.28	-4.67	-4.67	-4.67	**4.59**	1.42	-4.6
Potri.T029200	KTI	0	0	0	**4.73**	-0.19	0.95	**8.92**	**5.42**	-4.59
Potri.001G328000	WRKY 75	-0.26	0.34	**2.61**	0.59	1.36	**2.43**	0.17	0.46	**3.39**
Potri.T043800	WRKY 75	0.13	-0.24	**2.13**	1.08	1.7	**3.8**	-0.57	-6.83	**3.86**
Potri.003G169100	WRKY 75	0.51	0.89	1.25	1.39	1.22	**1.82**	-0.12	-0.77	**2.67**
Potri.015G099200	WRKY 75	-0.55	-0.31	**3.89**	0.15	0.34	**3.05**	0.7	-1.34	1.1
Potri.017G079500	WRKY 72	2.24	1.04	2.17	**3.62**	2.28	**4.08**	2.39	1.42	**10.64**
Potri.015G064100	WRKY 72	0.31	0.53	0.53	1.35	1.23	**1.84**	-0.26	0.35	**2.64**
Potri.001G352400	WRKY 71	0.6	0.01	0.81	1.48	0.85	**1.82**	0.88	0.47	**1.72**
Potri.013G090300	WRKY 70	0.58	0.63	**2.7**	-0.03	1.13	**1.85**	-4.1	-0.85	**1.87**
Potri.006G109100	WRKY 70	1.3	1.37	**2.41**	-0.06	0.94	1.48	-0.61	0.02	**2.4**
Potri.004G007500	WRKY 6	0.26	0.45	0.3	**1.99**	1.63	**1.87**	0.74	0.42	**2.23**
Potri.007G079800	WRKY 51	-1.24	-1.88	**2.82**	-1.15	0.13	0.89	0.33	-6.69	**4.43**
Potri.002G186600	WRKY 47	0.83	0.67	**2.57**	1.8	**1.98**	**3.25**	0.09	0.29	**2.76**

Selection of poplar Kunitz-type trypsin inhibitor and WRKY family genes expressed in each poplar species following inoculation with *Sphaerulina spp*. Numbers in bold show statistical significance at FDR < 0.05.

A number of different transcription factors showed differential expression throughout the infection. Of note were WRKY domain containing transcription factors that were differentially expressed in all pathosystems at 15 DAI (Table 4). Overall, more than one gene with significant expression mapped to each of the WRKY75, WRKY72, and WRKY70 families. However, overexpression of WRKY72 genes was not detected in *P*. *deltoides*.

### Comparison of RNA-seq analysis to RT-QPCR

**Fig 6:**
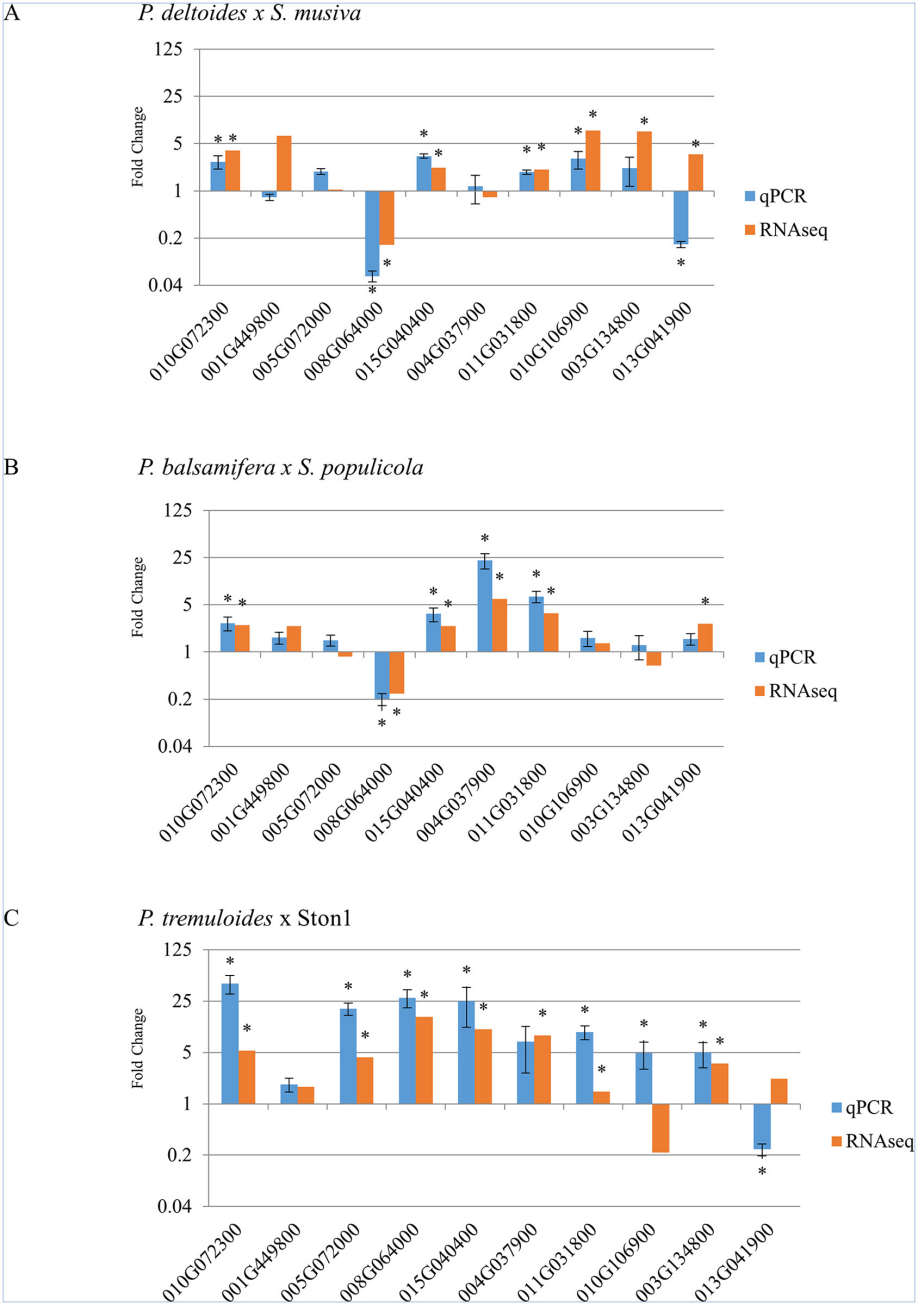
Comparison of gene expression levels between RNA-seq and reverse transcript qPCR by comparing gene expression at 0 and 15 DAI. (A) *P*. *deltoides* infected with *S*. *musiva*, (B) *P*. *balsamifera* infected with *S*. *populicola* and (C) *P*. *tremuloides* infected with Ston1. Labels indicate the *P*. *trichocarpa* v3 gene ID. Error bars show standard deviation. * represents significant difference in gene expression between 0 and 15 DAI with RT-qPCR measured by paired t-test at p < 0.05 and RNAseq measured by EdgeR at FDR < 0.05.

RT-qPCR was conducted using a small subset of genes for comparison between two time points: 0 and 15 DAI. Of the ten genes examined, seven genes followed the expected pattern of upregulation, downregulation or no change; two genes (Potri.001G449800 and Potri.010G106900) showed expression patterns different than expected in one poplar species, and one gene (Potri.013G041900) showed large inconsistency in observed gene expression in two pathosystems (Fig 6). All three genes that showed inconsistent expression between RNA-seq and RT-QPCR have close sequence similarity to other members in their respective gene family in *P*. *trichocarpa*. As such, it is possible that transcripts from other gene family members were co-amplified. All primers were designed using the *P*. *trichocarpa* reference sequences, but the use of more accurate species specific template libraries for primer design may allow for more precise RT-QPCR results. The lack of correspondence between RT- qPCR and RNAseq analysis may also be due to errors in read mapping related to the use of a non-species specific genome template or other issues generated with this data analysis pipeline.

## Discussion

The two unique host responses to leaf spot infection that observed. The first was the differential expression of many lignin biosynthesis in genes in *P*. *deltoides*. Lignification of cell walls surrounding sites inoculated with the Dothideomycetes pathogens was reported following *Zymoseptoria tritici* and *Phaeosphaeria nodorum* infection in wheat [32] and *S*. *musiva* stem canker in hybrid poplar [33,34]. During *S*. *musiva* canker formation on hybrid poplar, several layers of ligno-suberin necrophylactic periderm can form between the hyphal colonized tissues [32,34]. The presence or absence of these tissue layers may represent an accurate marker for disease resistance in hybrid poplar [34]. Susceptible hybrid clones were not observed to produce a necrophylactic periderm, while these barriers were observed to form rapidly in resistant trees inoculated without pre-wounding [34]. Other transcriptome studies in poplar have detected genes involved in lignin biosynthesis in response to wounding [35] and rust infection by both *M*. *larici-populina* and *M*. *medusa* [15]. However, no significant expression of genes directly involved in lignin biosynthesis was reported in either resistant or susceptible hybrid poplars in response to *S*. *musiva* leaf infection [19], though the upregulation of laccases encoding genes (diphenol oxidases) was observed. Laccases were also observed to be upregulated in all pathosystems at 15 DAI.

Senescence and abscission of diseased leaves in *P*. *balsamifera* was a unique phenotype. Genes putatively involved in senescence were overexpressed early in infection prior to the occurrence of any visible leaf symptoms. Leaf senescence and abscission in response to disease caused by *Drepanopeziza populi* and *M*. *medusae* was reported in another poplar species, *P*. *angustifolia*, particularly if plants were pre-treated with *Penicillium* spp. endophytes [8,36]. In blueberry, leaf senescence and abscission were reported following severe leaf infection by the *Sphaerulina* relative *Septoria albopunctata* [37]. In these studies, the pathogens caused leaves to senescence before abscission. Leaf abscission could be an indicator of severe infection or a triggered difference response. Within a natural forest setting, senescence and abscission of infected leaves early in the growing season could prove to be beneficial by removing a source of inoculum. Though leaf senescence and abscission was not observed in *P*. *deltoides* and *P*. *tremuloides*, some senescence-related genes were significantly overexpressed at later stages of infection.

The timing of necrotic spot appearance was fairly consistent between the different pathosystems, ranging from 8 to 14 DAI. This is within the range of necrotic leaf spot formation reported for various plant species infected with other Mycosphaerellaceae family pathogens. Previous studies have indicated the appearance of significant symptoms at approximately 20–32 days in wheat and blueberry following inoculation with *Z*. *tritici* and *S*. *albopunctata*, respectively [37,38]. Also, leaf spot symptoms on hybrid poplar leaf discs inoculated with *S*. *musiva* became visible in as early as 6 days [39]. Different inoculation conditions or differences in disease epidemiology may account for these observed differences.

Major differences in gene expression were observed in host gene expression of three poplar species during leaf spot infection by co-evolved *Sphaerulina* pathogens. However, RNA-seq data from infected leaves did show some common trends among the large differences in observed phenotypes. One common observation between all species was the expression of genes involved in defensive reactions early during infection, particularly chitinases and β-glucosidases (Table 3). Chitin has a primary role in pathogen detection in plants [40] and the upregulation of chitinase genes was previously reported after inoculation of hybrid poplar with the rust pathogen *M*. *medusae* [17]. Chitinases were also reported to be upregulated in hybrid poplar leaves 4 DAI with *S*. *musiva* [19]. In both *P*. *deltoides* and *P*. *balsamifera*, two β-glucosidase were identified as being moderately upregulated, indicating the possible start of cell wall modifications to defend against the invading pathogens. β-glucosidases are known to activate a group of compounds known as ‘phytoanticipins’, which are inert chemical defenses [41].

Kunitz trypsin inhibitor encoding genes are another group of defense related genes that were upregulated following challenge by *Sphaerulina* leaf pathogens in their poplar hosts (Table 4). Kunitz protease inhibitors can protect plant cells by blocking the action of pathogen proteases [42]. These genes were shown to be highly upregulated in hybrid poplar 24 h following wounding or insect herbivory [35,43,44]. Leaf infection of hybrid poplar by *M*. *medusae* induced a small upregulation of two Kunitz trypsin inhibitors at 1 DAI; however, these genes were downregulated at later stages of infection as the pathogen began to heavily colonize the leaves [17]. The early expression of trypsin inhibitors may indicate that cell damage occurred early in infection, though this was not quantified in this study.

Differential expression of many transcription factors was detected. WRKY family transcription factors were expressed by all three poplar species. A gene (Potri.015g099200) with similarity to *Arabidopsis* WRKY75 was significantly upregulated at 15 DAI in all three host species (Table 4). WRKY75 is involved in defense against both bacterial and fungal pathogens [45], and in *Arabidopsis*, loss of function for this gene showed delayed leaf senescence [46]. Overexpression of a single WRKY70 gene was also detected in all pathosystems (Potri.013G090300). In *Arabidopsis*, WKRY70 was identified as a key gene used in modulation of plant defense responses through suppression of jasmonic acid signalling [47]. These genes may play an important role in resistance to *Sphaerulina* pathogens and as the inducers of leaf abscission in *P*. *balsamifera*.

The functional annotations of differentially expressed genes revealed different hormone signalling pathways being activated in the different pathosystems. Ethylene signalling was observed in *P*. *deltoides*, and jasmonic acid and salicylic acid signalling were observed in *P*. *balsamifera*. Multiple hormone pathways were activated in *P*. *tremuloides*. Changes in hormone signalling gene expression were typically observed at 15 DAI with only ethylene signalling being detected in *P*. *tremuloides* during early infection. Some possible links can be hypothesized between hormone signalling and the observed plant phenotypes. Ethylene is known to play an important role in the inducible production of lignin [48], while jasmonic acid signalling is known to induce terpenoid production in plants [49]. The activation of these signalling pathways correlates with the observed upregulation of lignin and terpenoid biosynthesis genes in the different pathosystems. Different hormones have been implicated in defense against different pathogen classes. Ethylene and jasmonic acid signalling are typically thought to be involved in the defense against necrotrophic pathogens, while salicylic acid is an essential signalling hormone for defense against biotrophic and hemi-biotrophic pathogens [50,51]. The activation of different hormone signalling pathways may be an indicator of the hemi-biotrophic *Sphaerulina* pathogens at different stages of their infection cycle.

The data presented here shows an in depth examination of gene expression in different poplars with leaf spots induced by the different *Sphaerulina* species. Intraspecific variation in disease resistance was not examined; instead, what is presented here is a snapshot of the possible gene expression during *Sphaerulina* leaf spot infection in poplar. A number of commonly expressed genes were identified between the species examined among some large differences observed in whole pathway analysis and the associated plant phenotypes. The libraries generated here will be valuable for future studies. One limitation of this study was the use of the *P*. *trichocarpa* genome, as the poplar species we studied have different degrees of evolutionary distance from the model tree. A re-examination of this data may be conducted in the future when the precise genomes of these poplar species are completed and publically released and may also be utilized to annotate genes in future genome studies. An in-depth analysis of pathogen gene expression is also currently underway and will be published separately.

## Supporting Information

S1 Fig Multi-dimensional (MDS) scaling plot to visualize distances between RNAseq libraries. Ovals are estimates of variation between the biologically replicated libraries. Data points were computed using the 500 genes with the largest variation between the libraries. Distance between each data point is the square root of their common dispersion.
(TIF)

S2 Fig Venn diagrams showing differentially expressed upregulated and downregulated poplar genes from RNA-seq data mapped to the *P*. *trichocarpa* genome. Libraries from each time point are compared with the healthy, non-inoculated control.
(TIF)

S3 Fig Venn diagrams showing common upregulated and downregulated significantly expressed poplar genes common to the three pathosystems at each time point after inoculation. Libraries from each time point are compared with the healthy, non-inoculated control.
(TIF)

S4 Fig Fisher’s Exact Test of GO terms from *P*. *deltoides* assigned to differentially expressed genes overrepresented at 15 days following inoculation. No GO terms were overrepresented at 1 DAI or 4 DAI.
(TIF)

S5 Fig Fisher’s exact test of GO terms from *P*. *balsamifera* assigned to differentially expressed genes overrepresented at (A) 1 and (B) 15 days following inoculation. No GO terms were overrepresented at 4 DAI.
(TIF)

S6 Fig Fisher’s Exact Test of GO terms from *P*. *tremuloides* assigned to differentially expressed genes overrepresented at (A) 1, (B) 4 and (C) 15 days following inoculation. Only the top 25 GO terms are shown out of the 37 identified in (C). PCD = programmed cell death.
(TIF)

S1 Table List of primers used in this study for validation of RNAseq data and measurement of fungal growth by relative quantification of plant and fungal genes. (DOCX)

S2 Table Individual statistics for read mapping to *P*. *trichocarpa* genome. (DOCX)
