# Supplementary figures and images for: Transcriptome Analysis of Poplar during Leaf Spot Infection with Sphaerulina spp
Source: PLoS One. 2015 Sep 17;10(9):e0138162. doi: 10.1371/journal.pone.0138162 (PMC4575021; doi:10.1371/journal.pone.0138162)

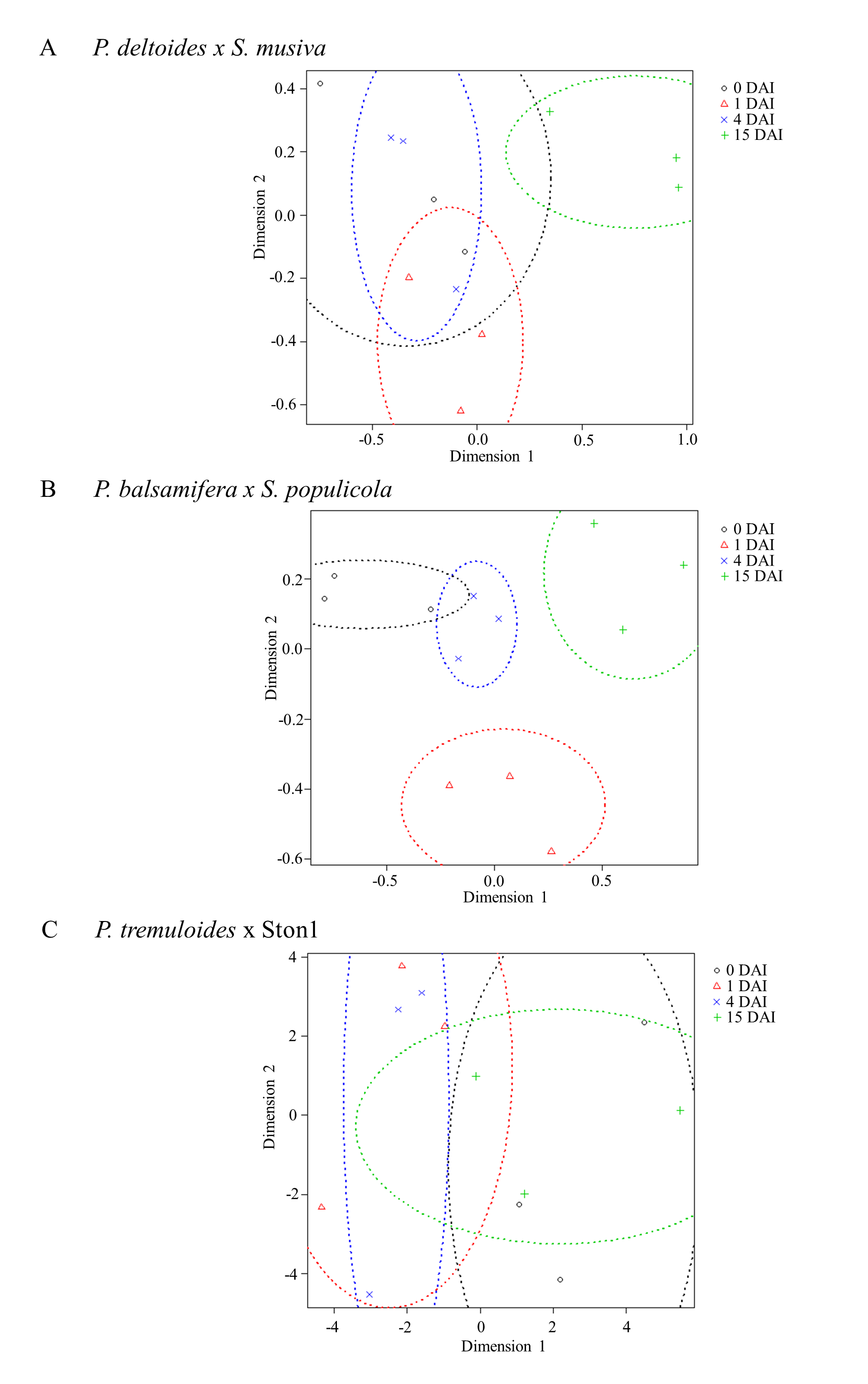

Supplement: S1 Fig — Ovals are estimates of variation between the biologically replicated libraries. Data points were computed using the 500 genes with the largest variation between the libraries. Distance between each data point is the square root of their common dispersion. (TIF) [file pone.0138162.s001.TIF]

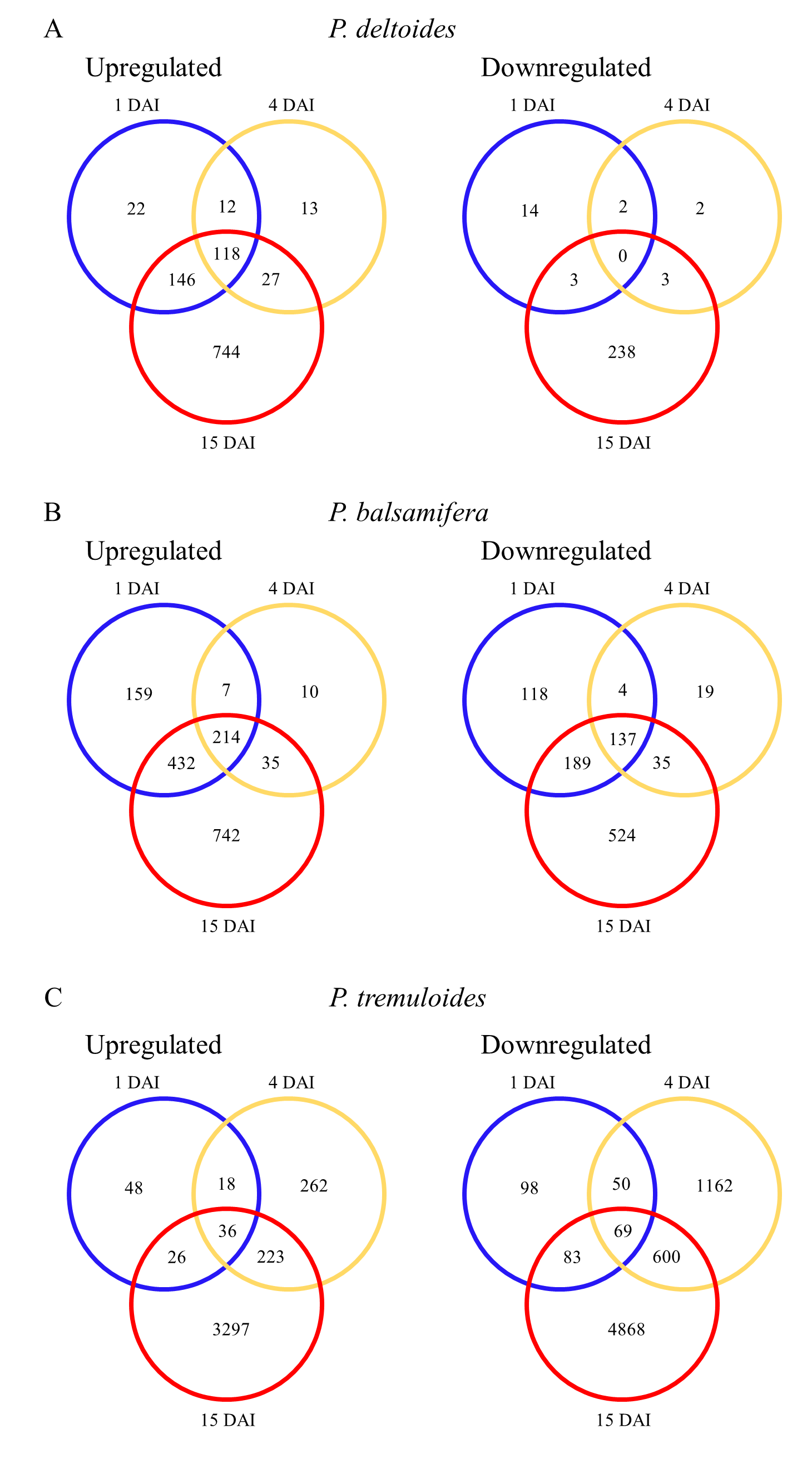

Supplement: S2 Fig — Libraries from each time point are compared with the healthy, non-inoculated control. (TIF) [file pone.0138162.s002.TIF]

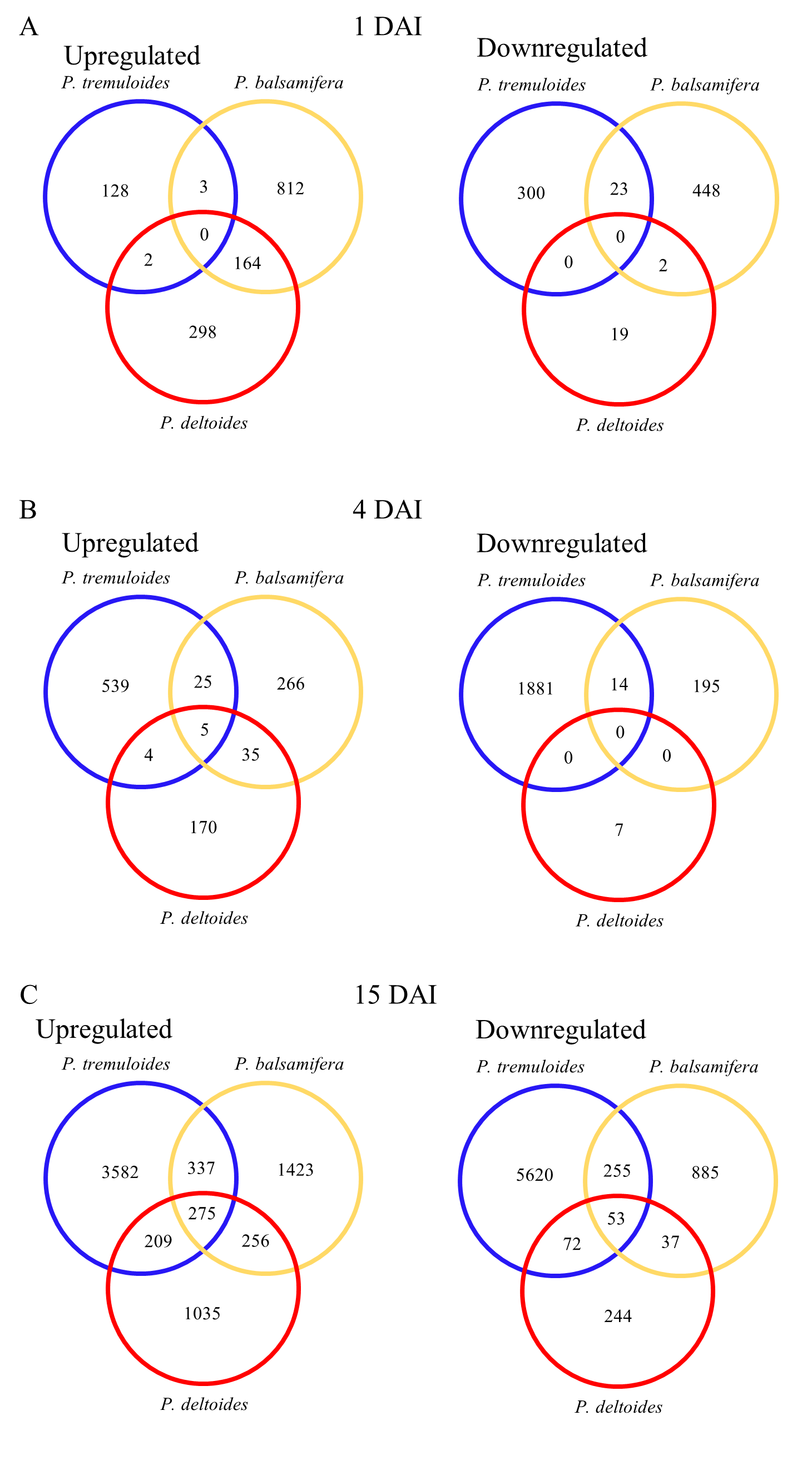

Supplement: S3 Fig — Libraries from each time point are compared with the healthy, non-inoculated control. (TIF) [file pone.0138162.s003.TIF]

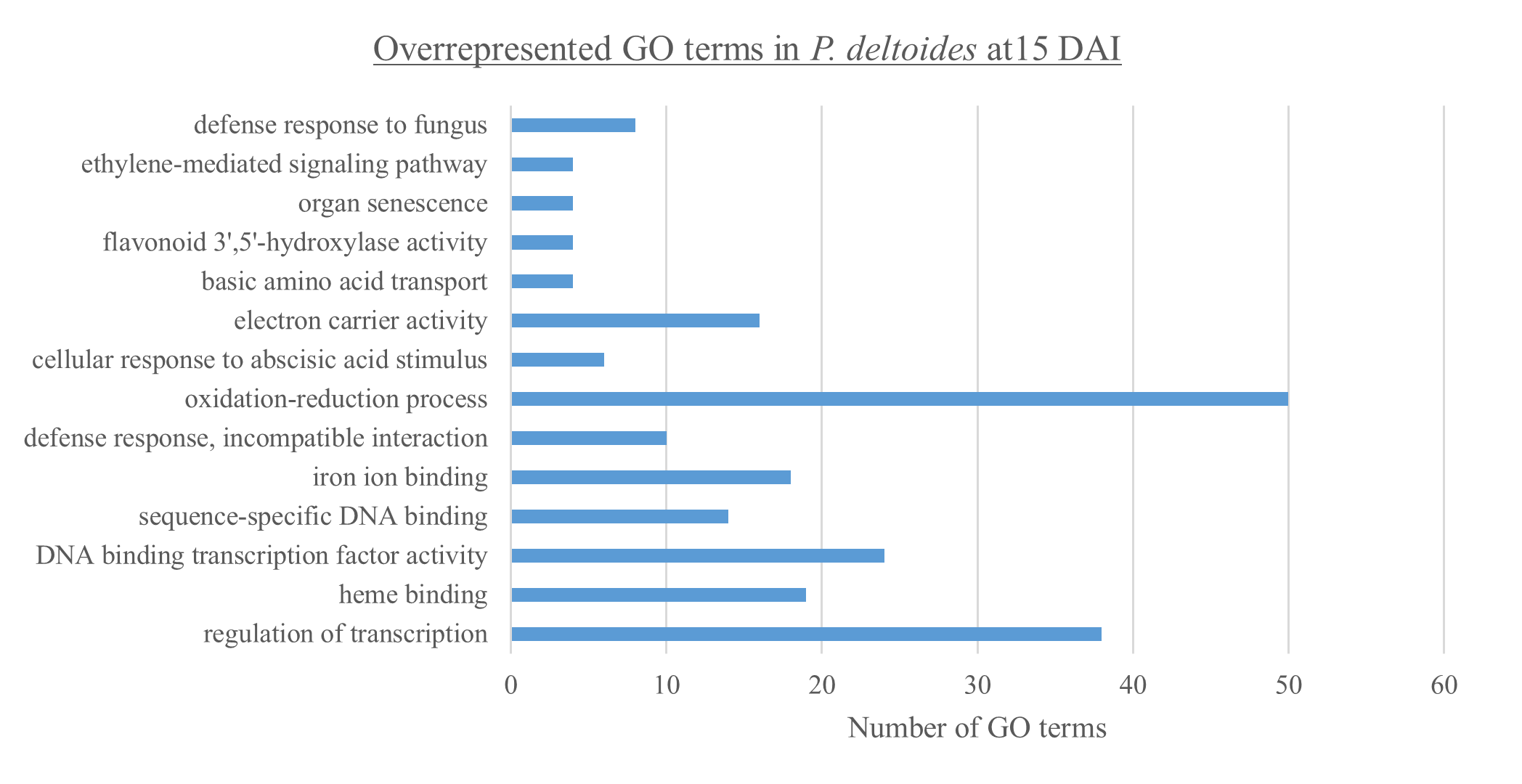

Supplement: S4 Fig — No GO terms were overrepresented at 1 DAI or 4 DAI. (TIF) [file pone.0138162.s004.TIF]

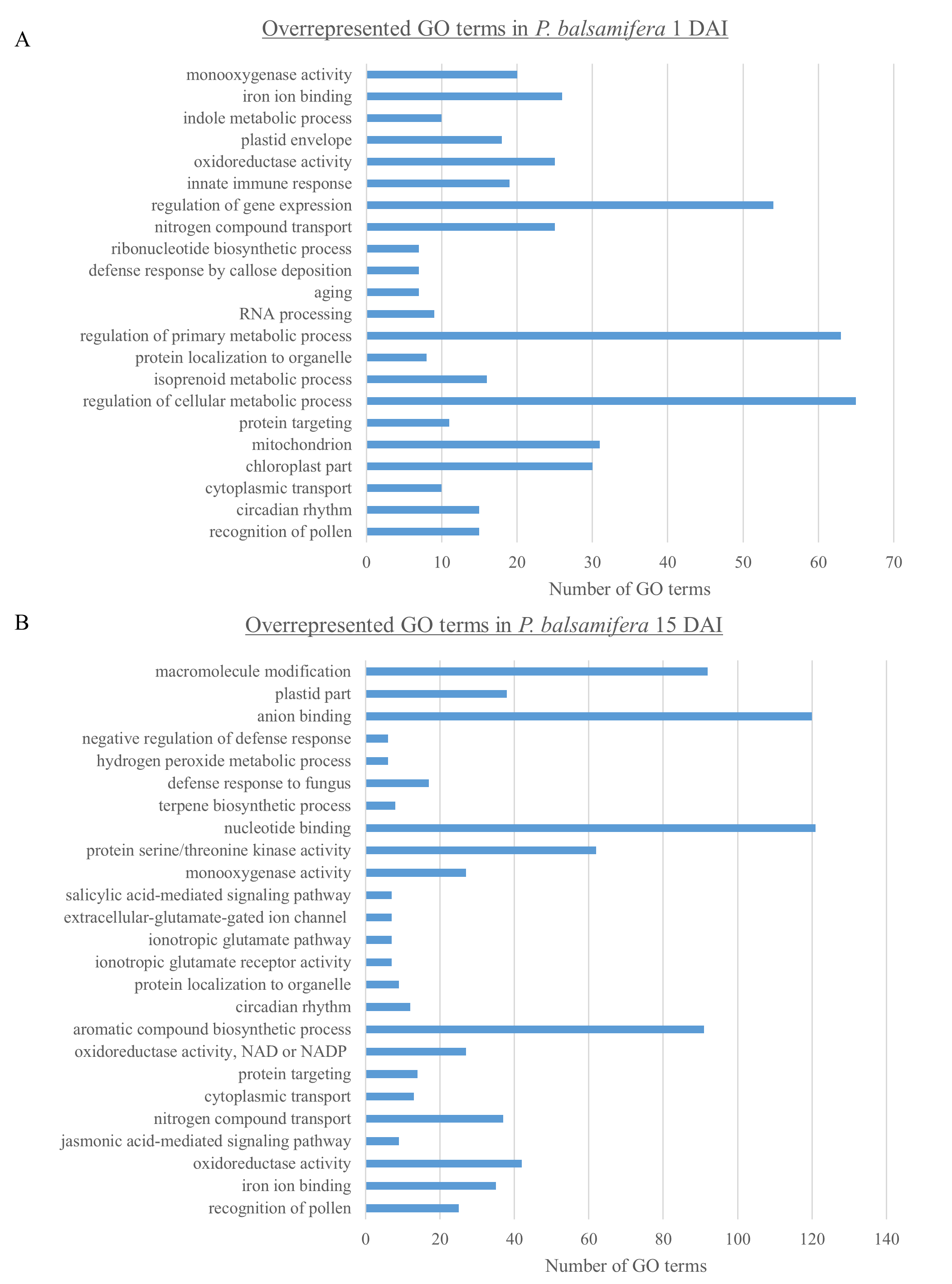

Supplement: S5 Fig — No GO terms were overrepresented at 4 DAI. (TIF) [file pone.0138162.s005.TIF]

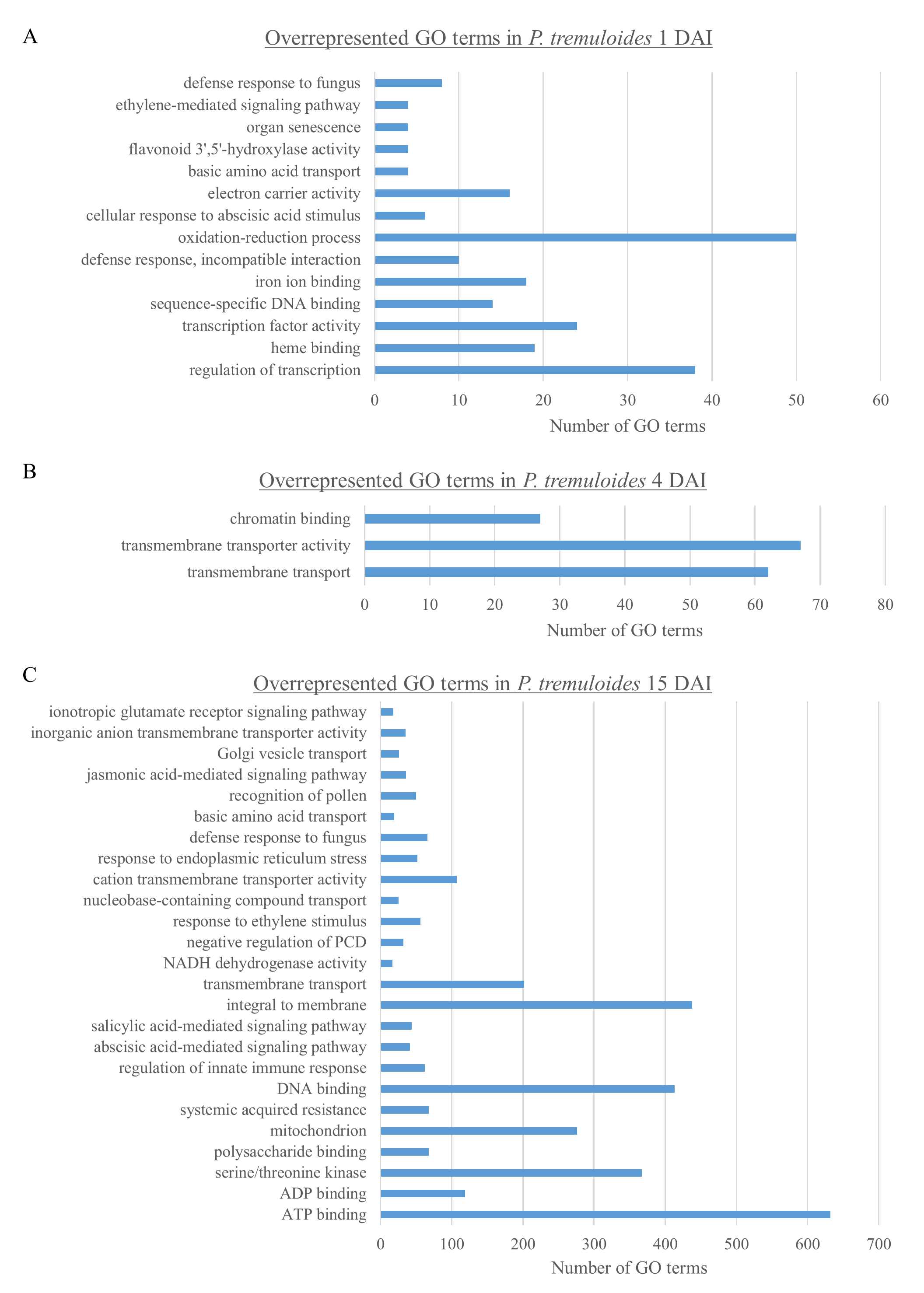

Supplement: S6 Fig — Only the top 25 GO terms are shown out of the 37 identified in (C). PCD = programmed cell death. (TIF) [file pone.0138162.s006.TIF]
