# Supplementary material for: Transcriptome Analysis of Poplar during Leaf Spot Infection with Sphaerulina spp
Source: PLoS One. 2015 Sep 17;10(9):e0138162. doi: 10.1371/journal.pone.0138162 (PMC4575021; doi:10.1371/journal.pone.0138162)
Supplement: S1 Table — (DOCX) [file pone.0138162.s007.docx]

**Table S1. List of primers used in this study for validation of RNAseq data and measurement of fungal growth by relative quantification of plant and fungal genes.**

| Target transcript ID | Species | Description | Primer ID | Sequence |
| --- | --- | --- | --- | --- |
| Potri.010G072300.1 | *P. tricocharpa* | ethelene response factor-1 | F545-570 | CAGTTGAAAGAGTGAGGGAGTCGCTT |
|  |  |  | R637-66 | GCTTCCCAATTTCCTTCTCATGGAGTA |
|  |  |  |  |  |
| Potri.001G449800.1 | *P. tricocharpa* | avr9 cf-9 rapidly elicited protein | F249-277 | ATGGATTGCAATACCAAGAGGGTTTTGAC |
|  |  |  | R329-350 | CAACGATCTCGAGGAGCTTCCC |
|  |  |  |  |  |
| Potri.005G072000.1 | *P. tricocharpa* | avr9 cf-9 rapidly elicited protein-2 | F69-103 | CGATCATCACAGAGAAAATACAAATCATGGGAGTT |
|  |  |  | R192-225 | GCTGATTGTAAAGTATTGACATAGATTAGCTGCG |
|  |  |  |  |  |
| Potri.008G064000.1 | *P. tricocharpa* | laccase | F1272-1301 | CTACTATAGCATAAGTGGGGTTTTCACAGA |
|  |  |  | R1342-1368 | ACTCTCAGGTGCTATTATAGTGGTTCC |
|  |  |  |  |  |
| Potri.015G040400.1 | *P. tricocharpa* | laccase | F1489-1516 | GTCACAACTTCTTCATTGTTGGGAGTGG |
|  |  |  | R1605-1628 | CTTGATCCTAATAGCAGCCCATCC |
|  |  |  |  |  |
| Potri.004G037900.1 | *P. tricocharpa* | s-linalool synthase | F1892-1920 | GGAGTGAAACATTTGCTTCCTGGTTTACG |
|  |  |  | R1957-1983 | CATGCCAGTCTCAAGATACTCTTCAGC |
|  |  |  |  |  |
| Potri.011G031800.1 | *P. tricocharpa* | sesquiterpene synthase | F188-208 | CACGGGTGGTCCATAACCTCT |
|  |  |  | R258-278 | ACAAGGAGGGTGAGGATCTGC |
|  |  |  |  |  |
| Potri.010G106900.1 | *P. tricocharpa* | lrr resistance protein | F1194-1219 | ACGTTGGGCAGCTTTTCAATCTTGAG |
|  |  |  | R1316-1344 | CAAGTGGGACGAAGATCAAGAACTAGTGA |
|  |  |  |  |  |
| Potri.003G134800.1 | *P. tricocharpa* | plant disease resistance response | F348-370 | ACCTAACCATCTTAGCAGGGCAG |
|  |  |  | R436-457 | CACCAGTTGGTAGGGCACAAGG |
|  |  |  |  |  |
| Potri.013G041900.1 | *P. tricocharpa* | wound induced (WIN1) | F143-166 | GAGGCCAAACCTGCGCTAACAATC |
|  |  |  | R217-247 | GACTACAGTTGCTTTGACAATTCTTGGAAGG |
|  |  |  |  |  |
| Potri.018G050800.1 | *P. tricocharpa* | protein kinase kinase | F401-426 | GCTAGAGATCCACCATCCATGTACTC |
|  |  |  | R475-501 | CAGTCATCACAATGCCCGTATGTTGTC |
|  |  |  |  |  |
| Potri.009G169500.1 | *P. tricocharpa* | alpha-expansin 4 | F225-249 | TCCCTTCACTTTCACAGCAATCACC |
|  |  |  | R322-344 | GTGAAGTGTGATAGAAGGGGTGG |
|  |  |  |  |  |
| Potri.006G225700.1 | *P. tricocharpa* | translation initiation factor eIF-4A | eIF4F1 | TGGGGCCTCTATTTAGCATGGAT |
|  |  |  | eIF4R1 | CTGCACCCGAAATGGGATTGACC |
|  |  |  |  |  |
| Potri.014G115100.1 | *P. tricocharpa* | ubiquitin/ribosomal protein | Ubiq10F1 | ACCAAGCCCAAGAAGATCAAGCA |
|  |  |  | Ubiq10R1 | CCAGCACCGCACTCAGCA |
|  |  |  |  |  |
| Potri.002G111900.1 | *P. tricocharpa* | α-tububulin-1 | α-tub1-F | TGTGGCTACCATCAAGACCA |
|  |  |  | α-tub1-R | TGTGGTCAATGCGAGAGAAG |
|  |  |  |  |  |
| Transcript 147624 | *S. musiva* | actin-1 | Smact-F | GACATTGGGACAGCTTCCAT |
|  |  |  | Smact-R | GGTGTGGTGCCAGATCTTCT |
|  |  |  |  |  |
| Transcript 133327 | *S. populicola* | actin-1 | Spact-F | AGCTTCCATTGTTGGACGAC |
|  |  |  | Spact-R | AGGTGTGGTGCCAGATCTTC |
|  |  |  |  |  |
| Contig00301:46340-46570 | Ston1 | actin-1 | Ston1act-r | GACAGAACCAGGACGAAGGA |
|  |  |  | Ston1act-F | CCGTGCTCAATTGGGTATCT |
