# Supplementary material for: Transcriptome Analysis of Poplar during Leaf Spot Infection with Sphaerulina spp
Source: PLoS One. 2015 Sep 17;10(9):e0138162. doi: 10.1371/journal.pone.0138162 (PMC4575021; doi:10.1371/journal.pone.0138162)
Supplement: S2 Table — (DOCX) [file pone.0138162.s008.docx]

**Table S2. Individual statistics for read mapping to P. trichocarpa genome**

| Plant | Fungus | DAI | Rep | Total reads | Reads mapped | % mapped | # genes |
| --- | --- | --- | --- | --- | --- | --- | --- |
| *P. deltoides* | *S. musiva* | 0 | 1 | 26558980 | 14446381 | 54.4% | 21997 |
| *P. deltoides* | *S. musiva* | 0 | 2 | 56576552 | 30489265 | 53.9% | 22827 |
| *P. deltoides* | *S. musiva* | 0 | 3 | 30919211 | 16898380 | 54.7% | 21485 |
| *P. deltoides* | *S. musiva* | 1 | 1 | 30988021 | 16454036 | 53.1% | 21312 |
| *P. deltoides* | *S. musiva* | 1 | 2 | 36762293 | 19827993 | 53.9% | 22053 |
| *P. deltoides* | *S. musiva* | 1 | 3 | 24351793 | 13074008 | 53.7% | 20574 |
| *P. deltoides* | *S. musiva* | 4 | 1 | 23013617 | 12562000 | 54.6% | 20762 |
| *P. deltoides* | *S. musiva* | 4 | 2 | 45698224 | 24969569 | 54.6% | 22456 |
| *P. deltoides* | *S. musiva* | 4 | 3 | 44876372 | 24259811 | 54.1% | 22306 |
| *P. deltoides* | *S. musiva* | 15 | 1 | 37989086 | 20826743 | 54.8% | 21743 |
| *P. deltoides* | *S. musiva* | 15 | 2 | 20223980 | 11022332 | 54.5% | 20487 |
| *P. deltoides* | *S. musiva* | 15 | 3 | 29357594 | 13663622 | 46.5% | 21525 |
| *P. balsamifera* | *S. populicola* | 0 | 1 | 21478434 | 12252901 | 57.0% | 21843 |
| *P. balsamifera* | *S. populicola* | 0 | 2 | 37945932 | 21646781 | 57.0% | 22892 |
| *P. balsamifera* | *S. populicola* | 0 | 3 | 22739512 | 12767135 | 56.1% | 21942 |
| *P. balsamifera* | *S. populicola* | 1 | 1 | 26248409 | 14748762 | 56.2% | 22072 |
| *P. balsamifera* | *S. populicola* | 1 | 2 | 24701544 | 13663622 | 55.3% | 21968 |
| *P. balsamifera* | *S. populicola* | 1 | 3 | 27305349 | 15174472 | 55.6% | 21882 |
| *P. balsamifera* | *S. populicola* | 4 | 1 | 28675221 | 15847347 | 55.3% | 22441 |
| *P. balsamifera* | *S. populicola* | 4 | 2 | 26639768 | 14432591 | 54.2% | 22617 |
| *P. balsamifera* | *S. populicola* | 4 | 3 | 32464547 | 17823687 | 54.9% | 22999 |
| *P. balsamifera* | *S. populicola* | 15 | 1 | 26656255 | 14554436 | 54.6% | 22414 |
| *P. balsamifera* | *S. populicola* | 15 | 2 | 45437538 | 24890378 | 54.8% | 23252 |
| *P. balsamifera* | *S. populicola* | 15 | 3 | 27939245 | 15377646 | 55.0% | 22541 |
| *P. tremuloides* | Ston1 | 0 | 1 | 15197762 | 7680506 | 50.5% | 21117 |
| *P. tremuloides* | Ston1 | 0 | 2 | 37201975 | 19322139 | 51.9% | 22810 |
| *P. tremuloides* | Ston1 | 0 | 3 | 38044360 | 18534589 | 48.7% | 22817 |
| *P. tremuloides* | Ston1 | 1 | 1 | 23256047 | 11294687 | 48.6% | 21878 |
| *P. tremuloides* | Ston1 | 1 | 2 | 29442427 | 13612097 | 46.2% | 22226 |
| *P. tremuloides* | Ston1 | 1 | 3 | 29266668 | 14330549 | 49.0% | 22716 |
| *P. tremuloides* | Ston1 | 4 | 1 | 9508273 | 4727899 | 49.7% | 19806 |
| *P. tremuloides* | Ston1 | 4 | 2 | 10541688 | 5282296 | 50.1% | 19942 |
| *P. tremuloides* | Ston1 | 4 | 3 | 37880709 | 12534005 | 33.1% | 16360 |
| *P. tremuloides* | Ston1 | 15 | 1 | 23909526 | 1803727 | 7.5% | 8968 |
| *P. tremuloides* | Ston1 | 15 | 2 | 32154610 | 14130990 | 43.9% | 21350 |
| *P. tremuloides* | Ston1 | 15 | 3 | 17699936 | 8699542 | 49.2% | 20876 |
| # genes is total number of genes identified with a minimum sum of 10 reads | | | | | | | |
